# Label-free robotic mitochondrial biopsy

**DOI:** 10.1126/sciadv.adx4289

**Published:** 2025-10-22

**Authors:** Yanmei Ma, Weikang Hu, Muyang Ruan, Feixiang Bao, Xingguo Liu, Dong Sun, Hongri Gu, Chengzhi Hu

**Affiliations:** ^1^ Shenzhen Key Laboratory of Biomimetic Robotics and Intelligent Systems, Department of Mechanical and Energy Engineering, Southern University of Science and Technology, Shenzhen, China.; ^2^ Department of Biomedical Engineering, City University of Hong Kong, Hong Kong, China.; ^3^ Guangzhou Institutes of Biomedicine and Health, Chinese Academy of Sciences, Guangzhou, China.; ^4^ Division of Integrative Systems and Design, Hong Kong University of Science and Technology, Hong Kong, China.

## Abstract

Robotic micromanipulation has advanced cellular probing, yet achieving precise, minimally invasive intracellular operations without fluorescent labeling remains challenging. Fluorescent techniques often cause photodamage and cytotoxicity and interfere with downstream analyses. Here, we introduce an automated, multifunctional nanoprobing platform capable of label-free extraction of mitochondria from living cells with high spatiotemporal resolution. The nanoprobe integrates two individually addressable nanoelectrodes that perform electrochemical detection of reactive oxygen and nitrogen species, produced by mitochondrial metabolism, followed by dielectrophoretic trapping, manipulation, and extraction of mitochondria. We successfully demonstrated the extraction of mitochondria from living cells, which is validated through fluorescence labeling before and after extraction. Subsequent quantitative polymerase chain reaction further confirmed that the extracted sample contained mitochondria. The fusion of the transplanted mitochondria within the recipient cell’s mitochondrial network confirms their activity. This automated, label-free, in situ organelle extraction micromanipulation system offers a powerful tool for understanding disease mechanisms linked to dysfunctional organelles and enables single-cell surgeries for organelle transplantation.

## Teaser

An automated nanoprobe system performs label-free mitochondrial sampling in living cells.

## INTRODUCTION

Since Hooke’s (*1*) first microscopic observation of cells in 1655, scientists and engineers have been driven to explore cellular structures and functions in ever-greater detail. This quest has led to the development of increasingly innovative tools designed to probe the cells. A milestone was the invention of the patch-clamp technique, which uses microelectrodes to record voltage signals across the cell membrane (*2*). This method directly contributed to the discovery of action potentials, laying the foundation of electrophysiology and earning the Nobel Prize in Physiology or Medicine in 1963 (*3*). However, probing individual cells remains inherently challenging because of their microscopic size, delicate nature, and low signal-to-noise ratio. Manipulating cells requires exceptional precision and gentle handling to preserve their viability and functionality during operations. Manually performed invasive measurements and manipulations can provoke unexpected cellular responses (*4*), complicating the interpretation of biological data and leading to inconsistencies and reproducibility issues across large samples (*5*).

The complexity of micromanipulation has sparked exciting opportunities for roboticists working to develop automated systems for more effective cell probing. This growing area, known as robotic micromanipulation, is reshaping cell biology by integrating advanced multifunctional microtools, high-precision imaging and motion control, and optimized system designs (*6*–*7*). Modern micromanipulation platforms have already demonstrated exceptional capabilities. For example, probe-based systems have evolved to include innovations such as nanotweezers for single-molecule trapping and extraction (*8*), atomic force microscopy–controlled nanopores capable of dynamic, high-resolution ion and molecule detection inside and outside of single cells (*9*), and FluidFM devices that enable continuous transcriptome profiling of individual macrophages (*10*). Continued progress in this area is bringing us closer to performing true “microsurgery” at the cellular level, enabling precise extraction, injection, dissection, and analysis of cellular components with unprecedented resolution and throughput. Looking forward, these technologies may even pave the way for assembling “artificial cells” by extracting organelles from different cells and reassembling them into entirely unique cellular entities—a concept reminiscent of a cellular “Frankenstein” (*11*).

To achieve precise micromanipulation inside individual cells, such as in biopsies, most systems rely on imaging fluorescent molecules attached to biological targets such as proteins or other cellular components. This approach allows for selective labeling of molecules of interest and enables real-time observation of dynamic biological processes within living cells. However, depending solely on fluorescent labeling presents major limitations for microrobotic manipulation. The primary challenge is spatiotemporal resolution. Accurate microrobotic manipulation at submicrometer scales requires both high spatial and temporal resolution to actively compensate for environmental disturbances, such as Brownian motion and cytoplasmic flow. Increasing the excitation light intensity to improve the resolution can cause photodamage to cells and alter their physiological dynamics (*12*). Therefore, for a given exposure dose, there is a trade-off between spatial and temporal resolution in experimental systems. In addition, fluorescent labeling can lead to photobleaching and cytotoxicity and may be incompatible with subsequent analytical techniques such as biochemical assays and gene editing (*13*). Some advanced imaging techniques require customizing the microscope, which may not be compatible with existing microrobotic manipulation systems, especially when a probe needs to be inserted into the cell (*7*).

A second challenge involves colocalization. In robotics, this classic problem involves aligning the coordinate systems of sensors and actuators to ensure that the signal measured at a specific position corresponds precisely to the point of manipulation. However, separating sensors and actuators makes colocalization particularly difficult, especially at submicrometer resolutions. Although highly accurate electrochemical sensors exist—such as those using silicon carbide nanotubes to detect reactive oxygen and nitrogen species (ROS/RNS) bursts from tiny vesicles in cancer cells (*14*)—these nanoscale sensors are not yet ready for integration into existing micromanipulation systems. When the micromanipulation probe moves inside the cell, it inevitably disturbs the local environment hydrodynamically, causing the target to drift away (*15*). In addition to spatial mismatches, the time delay between the sensor detecting the correct signal and the actuator moving to the correct position further reduces accuracy and decreases biopsy success rates.

Another major challenge is the lack of appropriate robotic platforms and protocols that can record, assess, and optimize the invasiveness of micromanipulation techniques. Cells are highly sensitive to physical parameters such as the nanoprobe insertion position, depth, and speed during cell penetration (*16*). These factors can profoundly influence various cellular activities, including migration, growth, differentiation, and programmed cell death (*17*). Manual intracellular manipulation of nanoprobes often results in unexpected cell death and poor experimental reproducibility (*18*). While most research focuses on interpreting biologically meaningful results, the impact of the micromanipulation systems used is rarely discussed. Although repetition and statistical analysis are common in cellular research, detailed information about micromanipulation procedures is often missing. This lack of a standardized framework makes it difficult to evaluate the invasiveness of robotic micromanipulation systems, especially when determining whether the observed effects are due to nonrepeatable micromanipulation or inherent heterogeneity within the cell population.

Mitochondria are essential organelles often referred to as the “powerhouses” of the cell because they generate most of the cell’s energy through aerobic metabolism. In addition to energy production, they are also major sources of ROS/RNS, which are by-products of mitochondrial metabolism. Under conditions of stress or inflammation, the levels of ROS/RNS rise substantially around mitochondria, which can profoundly affect cellular function and viability. This close link between mitochondria and ROS/RNS production provides a strong physiological rationale for precisely targeting and isolating mitochondria in studies of cellular function and disease (*19*). In this study, we present a label-free robotic micromanipulation system designed to overcome the aforementioned challenges by integrating both an electrochemical sensor and a dielectrophoretic (DEP) actuator at the tip of a nanoprobe. The electrochemical sensor is specifically engineered to detect ROS/RNS produced during mitochondrial metabolism. With high electrocatalytic sensitivity and a temporal resolution exceeding 1 kHz, the sensor allows us to quantitatively monitor the spatiotemporal production of ROS/RNS and track mitochondria in real time within living cells. The DEP nanotweezers effectively capture mitochondria within a range of 100 nm by exploiting differences in their dielectric properties. Our robotic system follows a well-designed protocol and collects associated data throughout the experiments—from cell detection, positioning the nanopipette tip in three dimensions near the target cell, detecting proximity between the tip and the cell surface, penetrating the cell membrane, recording electrochemical currents, and extracting mitochondria using dielectrophoresis based on electrochemical feedback. Because of the sharp tip and precise robotic control, the system has low invasiveness, allowing us to probe the same cell multiple times with a very high survival rate. We successfully demonstrated the transplantation of extracted mitochondria into other living cells, where they integrated into the host cell’s mitochondrial network and underwent fission. This work lays the foundation for advanced cellular micromanipulation techniques and provides a powerful tool for studying mitochondrial function in various cellular processes.

## RESULTS

### Fabrication and characterization of the multifunctional nanoprobe

**Fig. 1.**
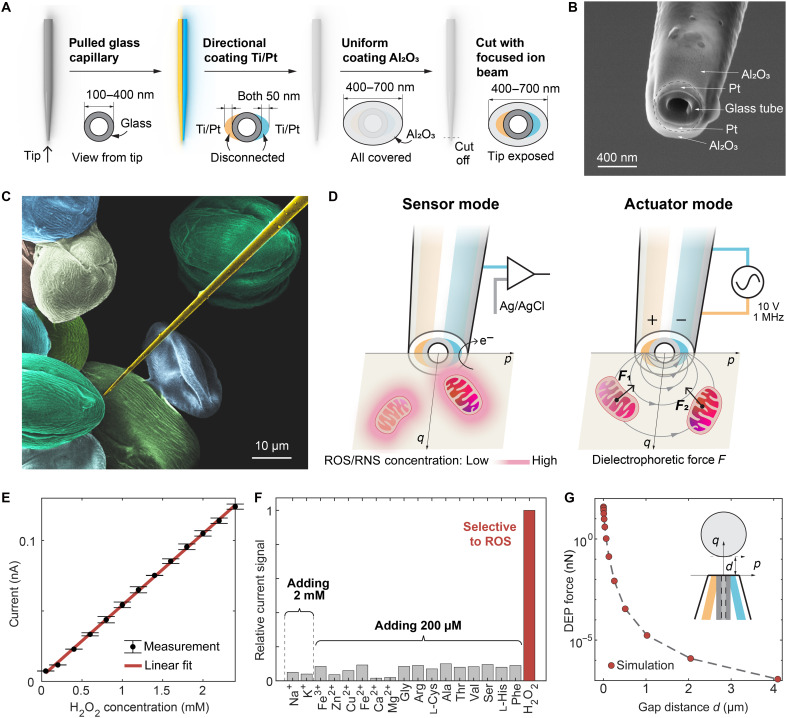
Nanoprobe structure, fabrication, and functions. (**A**) Schematic representation of the nanoprobe fabrication process: Starting with a capillary tube pulled to form a tip ~100 nm in diameter, the tip is coated with layers of 10-nm titanium (Ti) and 50-nm platinum (Pt). A 100-nm layer of aluminum oxide (Al_2_O_3_) is then applied for insulation. The insulation at the tip is precisely removed using FIB milling to expose the nanoelectrodes for experimental use. (**B**) Helium ion microscopy images showing the nanoprobe after the tip of the FIB milling process was exposed. (**C**) Color SEM image of the nanoprobe alongside pollen grains for size comparison. (**D**) Functional illustration of the nanoprobe: On the left, it acts as a ROS/RNS-sensing module to locate mitochondria based on intracellular ROS/RNS concentration gradients; on the right, it functions as an actuator module, extracting detected mitochondria through DEP forces. (**E**) Calibration curve showing the nanoprobe’s electrochemical response to various concentrations of hydrogen peroxide (H_2_O_2_) ranging from 0.05 to 2.4 mM. (**F**) Selectivity test results indicating the nanoprobe’s response to different species: 1 mM H_2_O_2_, 2 mM potassium (K^+^) and sodium (Na^+^) ions, and 200 μM other interfering substances. (**G**) Graph illustrating how the DEP force generated by the nanoprobe varies with the distance between the nanoprobe tip and a mitochondrion.

We fabricated the nanoprobe using a pulled glass capillary with a high aspect ratio (>22) and a tip diameter of less than 100 nm (Fig. 1A and fig. S2). Platinum-based metal electrodes were deposited onto the surface of the glass capillary. By exploiting the directionality of the electron beam deposition technique, two separate electrodes (indicated in blue and yellow in Fig. 1A) were created on opposite sides of the capillary without the need for a mask (fig. S3). To ensure electrical insulation from the external environment, we coated the entire nanoprobe with aluminum oxide. Last, the probe tip was precisely cut using a focused ion beam (FIB), exposing both the electrodes and the central lumen with a smooth, well-defined surface (Fig. 1B). A scanning electron microscopy (SEM) image of the probe over pollen grains (*Fritillaria unibracteata*), which are ~20 μm in size, highlights its slender geometry (Fig. 1C).

The platinum electrodes on the nanoprobe serve two primary functions (see Fig. 1D). First, they act as electrochemical sensors, leveraging the catalytic properties of platinum to selectively facilitate reactions with ROS/RNS under a small bias voltage (0.85 V). This oxidative reaction generates an electrochemical current (in about picoamperes) at the tip surface of the nanoprobe, which can be detected with an external amplifier. Second, the two separate electrodes function as a DEP nanotweezer. By applying a high-frequency alternating electric field (1 MHz) between the electrodes, cellular organelles such as mitochondria are attracted to the tip of the probe, where the electric field gradient is highest.

We conducted a comprehensive evaluation of the electrochemical sensor and DEP nanotweezer through experiments and simulations. According to the results of the calibration tests, the detected electrochemical current from the nanoprobe increases linearly with the concentration of the hydrogen peroxide solution (Fig. 1E and fig. S4), and the sensor’s performance is comparable to that of existing intracellular ROS/RNS sensors (table S1). As reported in previous studies, platinum electrodes exhibit high selectivity toward ROS/RNS compared with other common cellular substances (e.g., Na^+^, K^+^, and Mg^2+^) (Fig. 1F) (*14*). The nanoprobe demonstrated high stability, with its response current remaining unaffected by changes in pH (4.8 to 8) or high-speed probe motion (0.05 to 4 mm/s), and high spatial resolution of 1 μm in our testing setup (figs. S5, S6, and S7). In addition, numerical simulations of the DEP nanotweezer indicate that the force exerted can reach up to the nanonewton range when mitochondria contact the nanoprobe tip (refer to Fig. 1G, note S1, and figs. S8 and S9), comparable to that of other reported intracellular DEP nanotweezers (table S2) (*20*). The DEP force decays rapidly with increasing distance from the nanoprobe tip, providing high spatial selectivity and enabling mitochondrial trapping within ~1.8 μm around the tip (fig. S10 and note S2) (*21*).

### Complete process flow of automated robotic micromanipulation

**Fig. 2.**
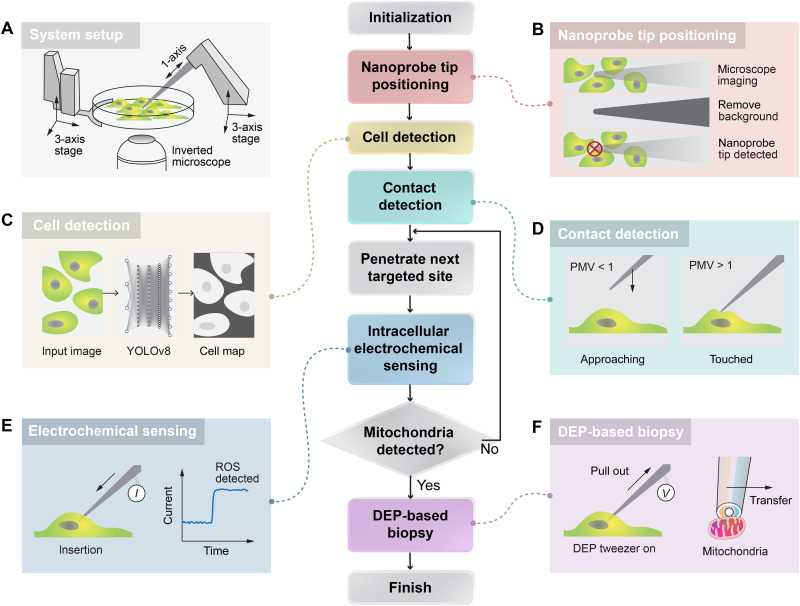
Automated robotic detection and manipulation of mitochondria. System workflow: The automated nanoprobe-based microoperation system performs three-dimensional (3D) positioning of the nanoprobe tip, detects cells, senses proximity between the nanoprobe tip and the cell surface, penetrates cells, and conducts intracellular electrochemical sensing to detect mitochondria. If mitochondria are detected, then they are biopsied using DEP forces. If not, then the system adjusts the penetration point and continues sensing until mitochondria are found and extracted, after which the program concludes. (**A**) System setup: The setup includes a three-axis translation stage for the cell platform and a four-axis micromanipulator, with the *D* axis aligned along the nanoprobe tip for cell penetration. An inverted fluorescence microscope provides imaging capabilities. (**B**) Nanoprobe tip positioning: This positioning is achieved through an enhanced background subtraction algorithm that isolates the nanoprobe tip in the microscope image by removing background elements. (**C**) Cell detection: A YOLOv8-based deep learning algorithm is implemented to identify and outline cells. (**D**) Contact detection: Using the motion history image algorithm, a pixel mean value (PMV) greater than 1 indicates contact between the nanoprobe tip and the cell membrane surface. (**E**) Electrochemical sensing: When the nanoprobe tip penetrates the cell, intracellular ROS/RNS react with the platinum at the tip, generating an oxidation current that signals the concentration of mitochondria. (**F**) DEP-based biopsy: An alternating voltage is applied to the nanoelectrodes on the nanoprobe, turning them into DEP tweezers that extract the detected mitochondria from within the cell.

Our robotic micromanipulation system is built around an inverted microscope and two three-axis motorized precision stages, allowing independent control of both the cell chamber’s position and the nanoprobe’s position (fig. S11). An additional degree of freedom enables the nanoprobe to move along its axial direction for insertion and extraction purposes. The complete process is illustrated in Fig. 2, with key steps graphically explained in colored tabs. Initially, the nanoprobe tip and the cells are detected separately, followed by vision-based contact detection to determine their relative positions. Using this information, the nanoprobe is inserted into the cell, and an electrochemical current is detected through the platinum sensor. If the current exceeds certain thresholds (8 pA), then we conclude that a mitochondrion has been detected. The DEP nanotweezer is then activated to trap the nearby mitochondrion and extract it from the cell, completing the biopsy. Further details are provided in figs. S12 to S16 and note S3 (*22*–*23*).

The integration of both the sensor and actuator at the tip of the nanoprobe greatly simplifies the manipulation process and enhances the dynamic responsiveness. When the nanoprobe tip senses an appropriate signal, it can immediately switch modes from sensor to actuator, allowing it to instantly trap nearby mitochondria. This contrasts with conventional solutions where sensors and actuators are separate; in these systems, the actuator probe must move to the position identified by the sensor, requiring precise colocalization. In our system, both transducers operate on the basis of electrical signals, offering fast dynamic performance (sensor rate, 1 kHz; actuator rate, 1 MHz), and the mode switch occurs instantaneously.

### Sensing ROS/RNS in single cells

**Fig. 3.**
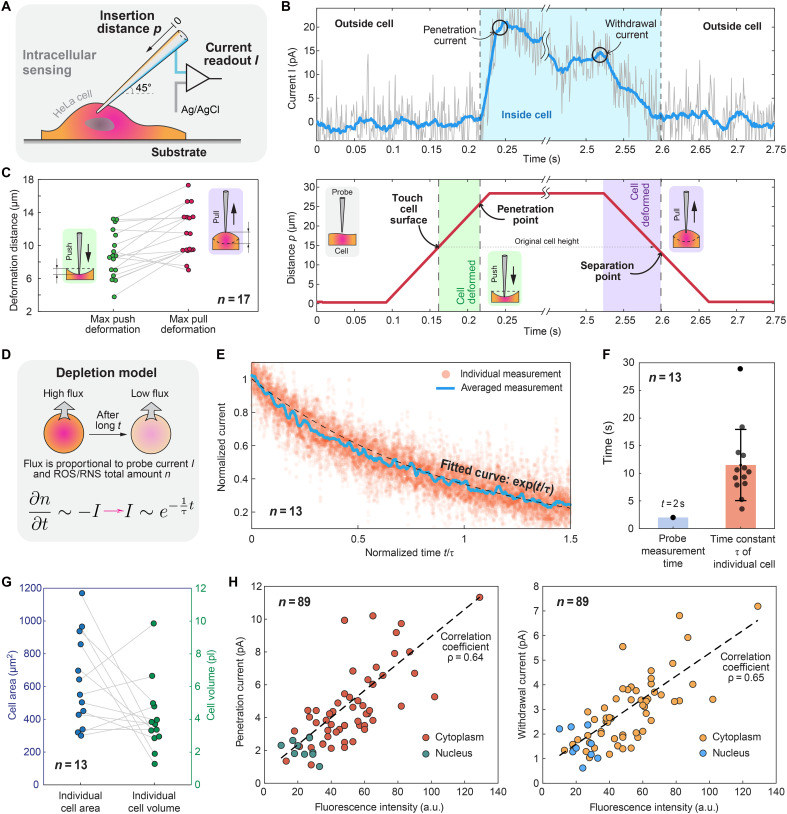
Detection of ROS/RNS in single cells. (**A**) Diagram illustrating intracellular sensing using the nanoprobe, which is fixed at a 45° angle on the micromanipulator. An Ag/AgCl reference electrode was placed in the solution to complete the electrochemical circuit. (**B**) Top: Current measurements during intracellular sensing. The gray line represents the raw data before filtering, whereas the blue line represents the data after filtering. The light blue–shaded area indicates the period when the nanoprobe is inside the cell. Bottom: Position data of the nanoprobe tip. The red line tracks the tip’s position, with the green area highlighting cell deformation during insertion and the purple area during retraction. (**C**) Statistical analysis comparing cell deformation during the nanoprobe insertion and withdrawal phases. (**D**) A depletion model illustrating changes in the ROS/RNS concentration near the nanoprobe tip during sensing. (**E**) Normalized current plotted against normalized time. The orange region represents individual measurements (*n* = 13), the blue line represents the average of these measurements, and the black dashed line represents the fitted curve. Time is normalized by dividing by the dissipation constant (*t*/τ). (**F**) Distribution of measurement durations and time constants from 13 intracellular sensing experiments. (**G**) Corresponding cell area and volume for each of the 13 measurements. (**H**) Correlations between the magnitude of the current measured during nanoprobe insertion (left) and withdrawal (right) and the mitochondrial fluorescence intensity within the cells. a.u., arbitrary units.

To measure intracellular ROS/RNS signals, the nanoprobe tip must penetrate the cell membrane to enter the cell interior. This penetration process is not always successful because it requires the nanoprobe to first deform the cell membrane and accumulate sufficient penetration force—typically ~10 to 100 nN—to achieve insertion (*8*). If the cell moves away during penetration, then the accumulated force dissipates, resulting in an unsuccessful attempt. In our study, we used adherent HeLa cells that grew attached to a substrate to prevent unwanted movement during penetration. Despite their adhesion, we observed instances where cells escaped during the automated penetration process. To increase the success rate, we positioned the nanoprobe at a 45° angle relative to the substrate, used a linear actuator to move the nanoprobe along its axial direction, and used a relatively fast insertion speed of 200 μm/s (Fig. 3A).

By integrating a sensor at the tip of the nanoprobe, we can directly measure the dynamics of cell membrane deformation by analyzing the sensor signals and nanoprobe position. As depicted in Fig. 3B, we observed a sudden increase in current during insertion. This spike serves as an indicator of membrane penetration since ROS/RNS concentrations are remarkably higher inside the cell. Before penetration, we performed contact detection (Fig. 2) to determine the relaxed position of the cell membrane. This allowed us to calculate the exact distance between the initial contact with the cell surface and the point of membrane penetration. Similarly, we measured membrane displacement when the nanoprobe was retracted from the cell. We assumed that after insertion, the membrane was fully relaxed after a 2-s measurement window. We defined a separation point where the current returned to zero and used the displacement up to this point as an indicator of cell membrane displacement during nanoprobe withdrawal, with the results shown in Fig. 3C.

Another observation was that the current measured did not remain constant but decayed over time. To understand this dynamic behavior, we conducted long-term measurements, and the results, presented in fig. S17, resembled an exponential decay. We explained this behavior using a simple depletion model (Fig. 3D). In this model, the cell is considered a sealed chamber with a fixed amount of ROS/RNS. When the nanoprobe is inserted, the electrochemical reactions at its tip consume ROS/RNS to generate an electric current, thereby reducing their concentration. The decreasing concentration further lowers the electric current, and this process continues until all the ROS/RNS are depleted (note S4 and fig. S17). This model allowed us to fit two parameters for each measurement: the initial ROS/RNS concentration (*C*_0_) and the characteristic time constant (τ), which are related to the cell volume (*V*) and electrochemical sensitivity (*A*) of the nanoprobe. We normalized our measurements by the decay time constant and the initial ROS/RNS concentration, as shown in Fig. 3E. The model closely matched our experimental results and enabled us to estimate individual cell volumes, which we compared to the areas of the same cells (Fig. 3G). The average time constant (τ) of this exponential decay process was ~12 s. Given our measurement window of 2 s, this decay has a relatively small effect on cell physiology (Fig. 3F).

An important question we explored was whether the platinum sensor detecting local ROS/RNS signals could determine the proximity of mitochondria, especially compared with conventional fluorescence imaging methods. We investigated the correlation between the ROS/RNS sensor signal and the mitochondrial concentration, quantified using the intensity of the local fluorescent signals, and the results are shown in Fig. 3H and fig. S19. The Pearson correlation coefficient was 0.65 for 89 individual measurements. Notably, we confirmed that the nuclear region presented both low ROS/RNS concentrations and low fluorescence signals, which is consistent with the findings of previous studies (*24*). Considering the complex membrane and cellular structures, we believe that the correlation is sufficiently strong to use the ROS/RNS signal as an indicator of the presence of nearby mitochondria.

### Characterization of DEP nanotweezers

**Fig. 4.**
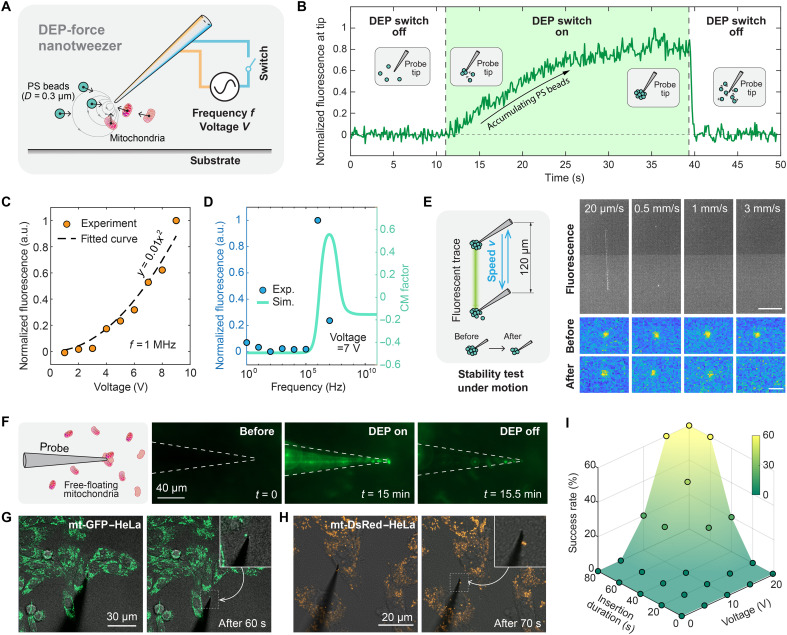
Functions and characterization of the DEP nanotweezer. (**A**) Schematic of the nanoprobe acting as a DEP tweezer to capture PS beads and mitochondria. (**B**) Graph showing the change in fluorescence intensity at the nanoprobe tip over time while trapping PS beads using DEP. The green-shaded area indicates the period when DEP is active (“DEP on”), during which PS beads accumulate at the tip, increasing the fluorescence intensity. This process is reversible; when DEP is deactivated (“DEP off”), the trapped beads are immediately released back into the solution. (**C**) Fluorescence intensity at the nanoprobe tip as a function of applied voltage at a constant frequency of 1 MHz. (**D**) Fluorescence intensity as a function of frequency at a constant voltage of 7 V. The cyan line represents the Clausius-Mossotti factor for mitochondria in a solution with a conductivity of 0.03 S/m. (**E**) Diagram showing changes at the nanoprobe tip during movement when PS beads are trapped. Movement can cause some beads to detach from the tip (left). Fluorescence images of the nanoprobe tip before and after movement at different speeds (right). Scale bars, 50 μm (top) and 5 μm (bottom). (**F**) Schematic of the nanoprobe trapping free-floating mitochondria, along with fluorescence images before DEP activation, after 15 min of DEP activation, and after DEP deactivation. (**G**) Fluorescence micrographs of green fluorescent protein–labeled mitochondria (mt-GFP)–HeLa cells and mt-DsRed–HeLa cells (**H**) during DEP biopsy (left) and after DEP biopsy (right). The successful extraction of mitochondria is indicated by fluorescent dots appearing at the nanoprobe tip following DEP manipulation. (**I**) Success rate of mitochondrial biopsy as a function of applied voltage and the duration of nanoprobe insertion.

We assessed the performance of the DEP nanotweezer using fluorescent polystyrene (PS) beads with an average diameter of 0.3 μm and a dielectric constant of 2.5, which are similar to those of mitochondria (*25*). Upon activation of the DEP nanotweezer, the surrounding PS beads were attracted by the DEP force and adhered to the nanoprobe tip. With the help of Brownian motion, additional particles move randomly and enter the trapping region (1.8 μm around the tip) dominated by DEP forces (note S1), leading to the formation of a cluster that grows until it reaches a maximum size. This dynamic accumulation process was observed through fluorescence imaging by monitoring the fluorescence intensity (Fig. 4B). When the DEP nanotweezer was deactivated, the attractive force vanished immediately, and the particle cluster disassembled within 1 s (Fig. 4B).

To further assess the performance of the DEP nanotweezers, we systematically varied the voltage and frequency of the applied oscillating electric field while monitoring the fluorescence signal as an indicator of trapping efficiency. As shown in Fig. 4 (C and D) and fig. S20, the fluorescence intensity increased quadratically with voltage, consistent with the well-established *V*^2^ dependence of the DEP force reported in previous studies (*26*–*27*). We determined the optimal operating frequency to be ~1 MHz. For subsequent experiments, a voltage of 7 V (equivalent to 0.3 mW) was selected to maintain efficient trapping while minimizing the risk of overheating at the nanoprobe tip (note S5 and fig. S21)

When the nanoprobe is moved through a solution, it must overcome fluidic drag forces, especially at relatively high speeds. For high-throughput and rapid robotic manipulation, it is important to determine the maximum speed at which the nanotweezer can stably hold the targeted objects. To address this, we moved the nanoprobe between two positions at different translation speeds after the nanotweezer was fully loaded with PS beads. As illustrated in Fig. 4E, the tip began to lose PS beads at speeds starting at 0.5 mm/s, and no fluorescent signals were observed at speeds of 3 mm/s. Building on the established performance results with PS beads, we tested DEP nanotweezers with free-floating mitochondria obtained through cell lysis (Fig. 4F) and within fluorescently labeled HeLa cells (Fig. 4, G and H). The overall success rate of intracellular mitochondrial extraction is summarized in Fig. 4I and fig. S22, which depends on the waiting time and the voltage of the driving signal (*f* = 1 MHz).

### Large-scale single-cell probing with an automated robotic system

To evaluate the overall performance of our automated robotic micromanipulation system, we conducted a large-scale systematic sensing operation on all cells within a specified area of interest identified from bright-field images. This assessment demonstrates the importance of a well-defined robotic system that records all micromanipulation data, which is essential for evaluating performance, assessing invasiveness, and guiding further system improvements.

**Fig. 5.**
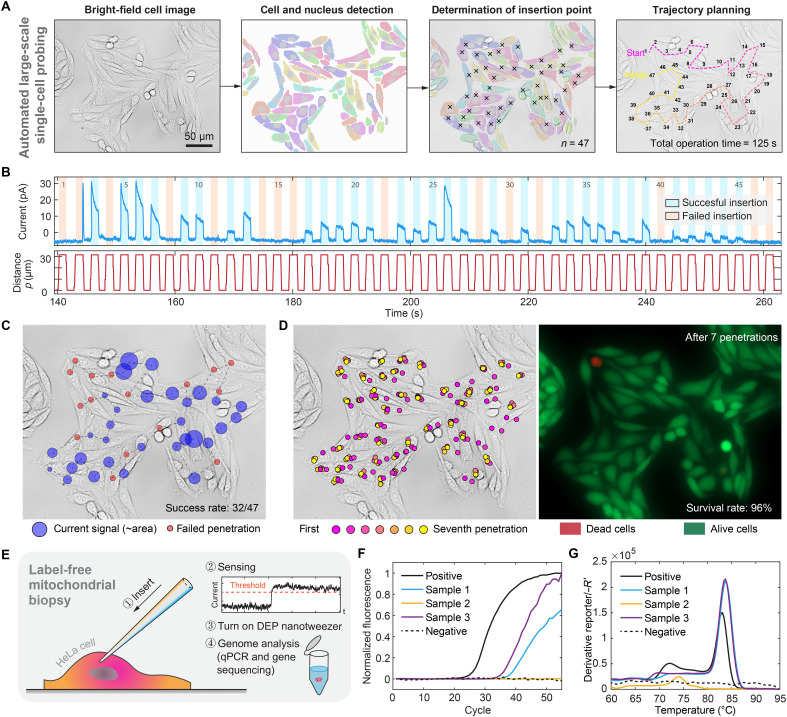
Automated large-scale single-cell ROS/RNS probing and survival rate analysis, combined with label-free mitochondrial biopsy in living cells. (**A**) Bright-field images of HeLa cells with detected cell contours and nuclei outlined using deep learning algorithms. The system determines the penetration points and plans paths accordingly. A total of 47 cells were targeted, and the entire operation took 125 s. (**B**) Top: Current measurements during cell penetration attempts. The blue areas represent successful penetrations, whereas the orange areas indicate failures. Bottom: Corresponding position data of the nanoprobe tip. (**C**) Visualization of the current magnitude for each cell, with the size of the purple circles proportional to the current measured. Red circles denote failed penetrations. The penetration success rate was 32 of 47 cells. (**D**) Distribution of penetration points for each cell over seven penetration cycles (left). Fluorescence images of HeLa cells stained with propidium iodide (PI) and calcein-AM (right). PI was added after the first penetration to identify dead cells, and calcein-AM was added after seven nanoprobe penetrations to assess cell viability. (**E**) Schematic diagram of the label-free biopsy of mitochondria. A nanoprobe is inserted into the cell for sensing. When the current exceeds a specific threshold, the nanoprobe acts as a nanotweezer to extract mitochondria by applying an ac voltage. The genomes of the isolated mitochondria are subsequently characterized qPCR and mtDNA sequencing. (**F**) Amplification curves obtained from the qPCR of the DEP-trapped mitochondria. (**G**). Sanger sequencing of the amplified sequence. The sequencing results show a near-perfect match between the extracted mtDNA (query) aligned with the corresponding mtDNA sequence (subject).

Within a densely packed cell cluster spanning an area of 0.62 mm by 0.5 mm, a total of 50 cells were identified using our computer vision program. Our algorithm automatically generated insertion positions for each cell, strategically avoided the nucleus, and optimized the probe trajectories (see Fig. 5A). After contact detection, the penetration depth was determined. To minimize physiological impact while sufficient ROS/RNS concentration data were collected, we programmed the probe to sense each cell for 1 s. The complete test for all *n* = 47 cells was finished within only 125 s. Up to this point, we fully recorded all aspects of the robotic micromanipulation with both the current signal and positions of the precision microstages (Fig. 5B), and we also recorded a video (movie S1). While this comprehensive data recording is common in robotics, it is rare in cellular research, where much information regarding the manipulation itself is often missing. This scarcity makes it challenging to evaluate processes and leads to reproducibility issues, and complete datasets, as we demonstrated here, can help.

On the basis of the jumps in the sensing signals, our automated robotic system successfully completed 32 of 47 probing operations (Fig. 5C). We observed that success rates depended on cell morphology; cells not fully attached to the substrate (with spherical shapes) tended to move during nanoprobe penetration, leading to failure. Notably, we found that the cell survival rate was very high (96%) (Fig. 5D). We performed additional nanoprobe testing after the first attempt, and most cells survived up to seven penetrations. This high survival rate indicates that our system’s invasiveness is minimal compared with other methods reported in the literature (table S3), and this is based on our complete testing of all cells in the same region, with all manipulation data recorded. We demonstrated a complete label-free biopsy using the onboard sensor and actuator to extract mitochondria based on the ROS/RNS current signal. Following biopsy, we performed quantitative polymerase chain reaction (qPCR) to confirm the presence of mitochondrial DNA (mtDNA) and further validated the successful extraction of mitochondria (see Fig. 5E). Specifically, the nanoprobe was automatically inserted into a living cell while monitoring the ROS/RNS signal. Once the current exceeded a threshold, DEP was activated for 60 s. The nanoprobe tip was then retracted from the cell while maintaining ac voltage to ensure complete extraction. The material was subjected to qPCR analysis, which revealed standard amplification curves that support the presence of mtDNA (Fig. 5F and table S4). Melting curves that were consistent with the target mtDNA were generated to confirm the specificity of the amplified product (fig. S24). Furthermore, Sanger sequencing revealed that the amplified sequence matched that of mtDNA (Fig. 5G). These findings confirmed that the mitochondria were successfully and specifically extracted. The functional integrity of the extracted mitochondria was further assessed by measuring mitochondrial membrane potential, and the biopsied mitochondria maintained J-aggregate formation, confirming their normal polarization state (fig. S25).

### Transplantation of mitochondria into living cells

**Fig. 6.**
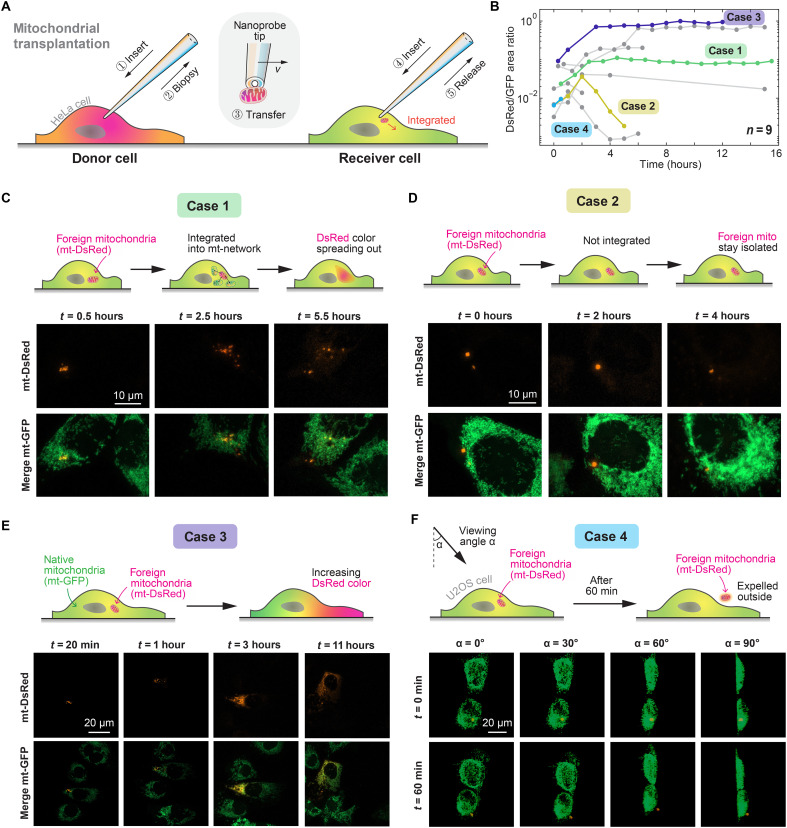
Robotic mitochondrial transplantation. (**A**) Diagram outlining the process of mitochondrial transplantation between living cells. Mitochondria are extracted from a donor cell (mt-DsRed–HeLa) via DEP biopsy. The nanoprobe containing the mitochondria is then moved to a recipient cell (mt-GFP–HeLa), where the mitochondria are released. (**B**) The ratio of red fluorescence (mt-DsRed) to green fluorescence (mt-GFP) areas at different time points after mitochondrial transfer across nine trials was used to evaluate the dynamic changes in the fusion and fission of mt-DsRed in mt-GFP–HeLa cells. (**C**) Case 1: Illustration showing the integration of foreign mitochondria (mt-DsRed) with the mitochondrial network of a recipient mt-GFP–HeLa cell. Fluorescence images of the recipient cell at various time points posttransplantation demonstrated the gradual integration of mt-DsRed into the cell’s mitochondrial network. (**D**) Case 2: Diagram depicting foreign mitochondria (mt-DsRed) failing to integrate into the recipient’s mitochondrial network. Fluorescence images of the recipient cell at various time points posttransplantation demonstrated that the number of transplanted mitochondria gradually decreased and the mitochondria remained isolated from the recipient cell’s mitochondrial network. (**E**) Case 3: Illustration showing the fusion of foreign mitochondria (mt-DsRed) with the mitochondrial network of a recipient mt-GFP–HeLa cell. Fluorescence images of the recipient cell at various time points posttransplantation demonstrated the gradual integration of mt-DsRed into the cell’s mitochondrial network. The continuous increase in red fluorescence may be due to mRNA being cotransferred during mitochondrial transplantation. (**F**) Case 4: Illustration of foreign mitochondria (mt-DsRed) in a recipient mt-GFP–U2OS cell, where the transferred mitochondria are expelled from the cell after 60 min. Fluorescence images from different angles provide visual confirmation of this process.

To validate the biological activity of the extracted mitochondria (marked with the red fluorescent protein, mt-DsRed), we directly transferred and injected them into HeLa cells with green fluorescent protein–labeled mitochondria (mt-GFP) (Fig. 6A). Within our nine independent trials, we observed diverse behaviors of the transplanted mitochondria in the receiver cells, some of which have not been previously reported (Fig. 6B). To explain the posttransplantation dynamics after our microrobotic manipulation system was used, we selected four examples as case studies, as shown in cases 1 to 4 in Fig. 6 (C to F). Our observations revealed that in six of the nine trials, the injected mitochondria were retained in the recipient cells after 1 hour, whereas in the remaining three trials, the mitochondria were lost. The loss of mitochondria could be attributed to clearance by cellular processes such as mitophagy or extracellular exocytosis. In case 4 (Fig. 6F), the transplanted mitochondria were expelled from the cell interior after 1 hour, which was confirmed by three-dimensional (3D) imaging via confocal microscopy.

Further examination of the red-labeled mitochondria within the recipient cells revealed that in four of nine trials, retained mitochondria successfully fused with the recipient’s mitochondria. This fusion was indicated by an increase in the area of red fluorescence and colocalization with the green-labeled mitochondria, with red-green overlap areas increasing over time (case 1 in Fig. 6C). However, some mitochondria remained distinct and failed to integrate into the recipient’s mitochondrial network (case 2 in Fig. 6D). This lack of fusion may suggest dysfunction, which is likely resolved gradually. Certain recipient cells exhibited unexpected increases in both the intensity and area of red fluorescence. As the mt-DsRed gene originates from the donor cell’s nucleus, mitochondrial transplantation led to a gradual decline in red fluorescence, given the lack of ongoing mt-DsRed transcription and translation in the recipient cells. However, in case 3 (Fig. 6E and fig. S26), we observed increased red fluorescence, which integrated well into the receiver cell mitochondrial network. This phenomenon may be attributed to the potential translation of mRNAs originating from the donor cytoplasm. The observed changes in fluorescence intensity might result from mRNA being cotransferred during mitochondrial transplantation, enabling temporary translation of mt-DsRed within the receiver cell. This finding suggests possibilities for combined cytoplasm and metabolite transplantation, offering a method to transfer not only target organelles but also donor metabolites and essential biomolecules to the recipient cell, potentially enhancing cellular function.

Our method advances mitochondrial transplantation by directly transferring mitochondria from donor to recipient cells under natural physiological conditions. Unlike conventional methods, which often require in vitro steps such as homogenization and centrifugation that expose mitochondria to external conditions and risk compromising their functionality (*28*), our approach avoids these disruptions through direct transfer between donor and recipient cells. By using an automated handling process, we minimize the exposure time and reduce the risk of functional deterioration, thereby maintaining biological integrity for the transplant. Previous in vitro extraction methods, in which mitochondria were successfully introduced into recipient cells, likely triggered indirect phenotypic effects primarily through mitophagy rather than through true mitochondrial integration (*29*). The external isolation of mitochondria can initiate cellular stress responses, including mitophagy, which may lead to the degradation of the introduced mitochondria. In our study, the observed fusion between donor and recipient mitochondria directly supports the feasibility of achieving mitochondrial integration. This finding not only underscores the robustness of the transplantation process but also suggests the potential for stable, long-term functionality of the transplanted mitochondria.

Considering that the fusion efficiency between donor and recipient mitochondria in our system is suboptimal, several limiting factors may be at play, including the autophagy of transplanted mitochondria and barriers to fusion between endogenous and exogenous mitochondria. To address these challenges, we could explore strategies to inhibit mitophagy and lysosomal activity, which may enhance the retention of transplanted mitochondria. Furthermore, pharmacological agents such as M1, which have been shown to promote mitochondrial fusion and facilitate processes such as embryoid body cardiac differentiation in human pluripotent stem cells (*30*), could be used to improve mitochondrial integration in recipient cells. These adjustments could create a more favorable environment for mitochondrial incorporation and enable deeper insights into the mechanisms driving mitochondrial transplantation.

## DISCUSSION

In this research, we present a fully automated robotic platform that enables label-free intracellular biopsy and mitochondrial transplantation in living cells. By integrating a sensor and an actuator at the tip of a nanoprobe, we achieve high spatiotemporal resolution without the need for colocalization techniques. This nanoprobe allows us to detect and extract mitochondria from live cells without relying on fluorescent labeling. Our micromanipulation system is minimally invasive, resulting in a very low cell death rate after detection (96% survival rate), and remained at a survival rate of 80% following DEP activation (fig. S23). We demonstrate that the extracted mitochondria remain viable and can be transplanted into other living cells, where they integrate with the recipient’s mitochondrial network and undergo fission.

A crucial aspect of our system is the size of the nanoprobe and its potential for miniaturization. The sensor must strike a balance between sensitivity and minimal impact on cell physiology. Smaller electrodes generate less current, which can reduce the sensitivity and make electrochemical signals harder to detect because of noise. However, a smaller surface area consumes fewer ROS/RNS, causing less disruption to cellular conditions and allowing for longer observation periods. Regarding the DEP nanotweezer, smaller electrodes produce weaker forces at the same distance, which may require more time to securely grasp the targeted organelle and could lower the success rate. This trade-off suggests that the tip geometry should be similar in size to the target organelle for optimal DEP actuator performance, even if other compromises are necessary.

In addition, the nanoprobe is adaptable and can be modified to include various electrochemical sensors capable of detecting additional cellular physiological signals, such as temperature, pH and Ca^2+^ levels (*31*–*33*). The intracellular distributions of these parameters are inherently heterogeneous. For example, mitochondria release heat during adenosine 5′-triphosphate production via oxidative phosphorylation and exhibit temperatures at least 2°C higher than those of the surrounding cytoplasm (*34*). Substantial pH differences are also observed among various organelles: While the cytoplasm maintains an average pH of 7.2, lysosomes exhibit a much lower pH of 4.8, and the mitochondrial matrix can reach a pH as high as 8.0 (*35*). In addition, the concentration of Ca^2+^ ions in the endoplasmic reticulum (10^−3^ M) is markedly higher than that in the cytoplasm (10^−7^ M) (*36*). These pronounced variations in analytes across different cellular compartments, combined with the use of diverse intracellular nanoscale sensors, enable label-free extraction of multiple components. Advanced sensors and actuators created through state-of-the-art flexible electronics and 3D printing technologies can also be integrated into nanoprobes (*37*), enhancing their sensing capabilities (e.g., molecule detection, force detection, and nanodiamonds) and actuation functions (e.g., magnetic, optical, and thermal), as well as expanding their application scope (e.g., 3D culture environments and suspension cell studies).

At present, we rely on qPCR to verify the extracted mitochondria because the sample from each run of our automated platform is insufficient for most existing single-cell omics workflows. Nevertheless, rapid advances in ultralow-input library preparation and single-cell multiomics make it technically feasible to work with picogram-level material (*38*). Because of its open, modular architecture, our platform could be seamlessly interfaced with single-cell DNA/RNA sequencing, proteomics, and even metabolomics instruments, thereby enabling subcellular-resolution multiomics analyses. In future work, we plan to (i) refine the extraction and purification steps to minimize sample loss and (ii) explore coupling with proteomic and metabolic assays to dissect mitochondrial function and its role in cell-fate decisions.

Focusing on mitochondria is particularly important because of their vital role in regulating cellular processes and their relevance to many diseases. Mitochondrial dysfunction is implicated in the development of various conditions, including metabolic disorders, aging, tissue injuries, tumors, and cancers (*39*–*41*). Disorders caused by mtDNA mutations, such as Leigh syndrome and mitochondrial encephalomyopathy, often exhibit mutation rates near 100% in affected cells. Heteroplasmic mutations frequently surpass the 70 to 90% pathogenic threshold, especially in hotspots such as the *MTND5* and *MTND6* genes (*42*). These mtDNA mutations display a threshold effect, with symptoms appearing only when mutations exceed a certain level (*43*). Our mitochondrial transplantation method offers a potential strategy to reduce mtDNA mutation rates by introducing healthy mitochondria into patient cells, effectively diluting the overall mutation burden. Furthermore, by combining this transplantation approach with techniques for mtDNA or mitochondrial depletion (*44*–*46*), there is promising potential to achieve complete replacement of mutated mtDNA. This combined strategy could potentially allow for the full replacement of mutated mitochondria.

Our microrobotic platform enables mitochondrial transplantation, unlocking exciting opportunities in biological research and cellular therapies. Introducing entire, healthy mitochondria into recipient cells is a straightforward yet highly effective therapeutic approach, showing great promise in treating various diseases (*47*). This technique is not only powerful on its own but also likely to inspire applications across multiple research fields. For example, it could be used to rejuvenate cells with diminished metabolic activity in stem cell therapies or serve as an alternative strategy for mitochondrial replacement therapy. In addition, it opens alternative pathways for investigating fundamental questions in cell biology, mechanobiology, and cell engineering (*48*). We are confident that our micromanipulation system provides a robust platform for analyzing single cells at different locations within a living organism and at various stages of cellular processes, such as movement and division. Its versatility allows it to be used in diverse environments, including mixed cell cultures, adherent primary cell probing, and during treatments such as chemotherapy or immunotherapy. By using this system, researchers can observe how individual cells evolve over time within a heterogeneous population, offering a precise tool for quality control in relevant therapeutic tests at the single-cell level.

## MATERIALS AND METHODS

### Materials and reagents

Borosilicate glass capillaries (BF-100-50-10) were purchased from Sutter Instruments (USA), and Ru(NH_3_)_6_Cl_3_, hydrogen peroxide (H_2_O_2_; 30 wt %), phosphate-buffered saline (PBS), and phorbol 12-myristate 13-acetate (PMA) were purchased from Aladdin Industrial Corporation (Shanghai, China). Minimum essential medium (MEM) was purchased from VivaCell (China). CO_2_-independent growth medium, fetal bovine serum (FBS), penicillin, and streptomycin solutions were purchased from Gibco (USA). Propidium iodide (PI) and calcein-AM were purchased from Thermo Fisher Scientific (USA). Fluorescent PS beads with a size of 300 nm were purchased from Beilesi (China). MitoTracker Green FM was purchased from Shanghai Yuanye Bio-Technology Co. Ltd. (China).

### Fabrication and characterization of nanoprobes

The nanoprobes were fabricated from borosilicate glass capillaries through a four-step process (fig. S1). Briefly, the capillaries were initially pulled into a sharp nanopipette using a one-line program on a P-2000 laser puller (Sutter Instrument, USA). Then, 10-nm Ti and 50-nm Pt layers were coated onto both sides of the nanopipettes by rotating 180° around their axis after the first deposition was completed (TF500, China). During this process, nanometer-sized gaps were created because of the existence of shadow regions perpendicular to the direction of deposition. The coated nanoprobe was then covered with Al_2_O_3_ as an insulation layer (R-200, China), and the final tip of the nanopipette was milled with an FIB (ORION NanoFab, USA) to expose the nanoelectrodes. Unless otherwise specified, all of the nanoprobes were manufactured immediately before the experiments and stored in a sealed dish to minimize contamination. Before intracellular penetration, we functionalized the nanoprobes with SL2 Sigmacote, a hydrophobic treatment that reduces nonspecific adsorption of biomacromolecules (*10*). The tips of the nanoprobes were characterized by SEM (ZEISS Merlin, Germany) coupled with an energy-dispersive spectrometer (Octane Pro).

### Cell culture and viability test

mt-GFP–HeLa and mt-DsRed–HeLa cells were provided by X. Liu from the Chinese Academy of Sciences and maintained in MEM supplemented with 10% fetal bovine serum, penicillin (100 IU/ml), and streptomycin (100 mg/ml). The cells were cultured in a humidified incubator at 37°C with 5% CO_2_. Fluorescence detection and cell insertion experiments were performed after the cells had adhered to the planar substrates. Calcein-AM and PI were added to the medium following the manufacturer’s protocols. In the survival rate experiment, PI was added after the first penetration at 30 min, and calcein-AM was added after seven runs of penetration with a nanoprobe. Bright-field and fluorescence microphotographs were captured using a charge-coupled device mounted on an inverted fluorescence microscope.

### Extracellular electrochemical detection of H_2_O_2_

All extracellular current measurements were recorded with an electrochemical workstation (CHI760E, CH Instruments Inc., Shanghai) in conjunction with a CHI-200B microcurrent signal amplifier. For the detection of H_2_O_2_ in the solution, a chronoamperometry measurement was performed by applying a potential of 0.85 V and adding a 10 mM PBS solution to the bath solution. After the background current stabilized, H_2_O_2_ was added to the bath solution to obtain final concentrations ranging from 0.05 to 2.4 mM. The current was continuously recorded to investigate the current response of the nanoprobe to H_2_O_2_. The concentration of each interference component was 200 μM to test the selective profile of the nanoprobe (K^+^ and Na^+^ were 2 mM). The stability of the nanoprobe was subsequently assessed through repeated tests of the nanoelectrode in the same solution.

### Intracellular electrochemical detection of ROS/RNS

All single-cell intracellular experiments were accomplished by our automated nanopipette-based microoperation system. The nanoprobe was anchored on the holder of a four–degree of freedom micromanipulator (μMp-4, Sensapex, Finland). The Ag/AgCl reference electrode was placed near the nanoprobe. The micromanipulators were equipped with an inverted microscope (Axio Observer 7, Zeiss, Germany) for precise control of the nanoprobe during its insertion into single cells under observation. The intracellular current measurements were recorded using Axopatch MultiClamp 700B low-noise amplifier (Axon Instruments, USA). The MultiClamp 700B amplifier was used in gap-free mode with the Digidata 1550 digitizer (Molecular Devices, USA) and a PC equipped with pCLAMP11 software (Molecular Devices). To induce oxidative stress inside the cell, PMA (1 μg/mL) was added to the petri dish to stimulate the cells for 30 min. The cells were then washed three times with PBS and replaced with fresh PBS for subsequent intracellular electrical signal collection. The intracellular current measurements were recorded at a constant potential of 0.85 V. The control group that does not receive any drug treatment was also tested using the same methods but without PMA stimulation (fig. S18).

### Mitochondrial fluorescence and corresponding current signal measurement

In Fig. 3H, the sensor signal and fluorescence signal were acquired as follows: First, the mitochondrial fluorescence image of the cell (fig. S19A) was captured. Then, specific insertion points were selected for the cell, and the current detected at these points (including the nanoprobe insertion current and withdrawal current) (fig. S19B) was recorded. Last, the relationship between the mitochondrial fluorescence intensity at the insertion point and the corresponding detected current was established, as shown in Fig. 3H.

### DEP trapping and manipulation of PS nanobeads

The ac bias was applied between the two nanoelectrodes of the nanoprobe via a function generator to generate the DEP trapping force (Tektronix AFG31000, USA). DEP trapping and manipulation of PS nanobeads were demonstrated using 300-nm fluorescent nanobeads suspended in deionized water. For the trapping experiments, a 10^5^-fold dilution of the nanobead solution was used to avoid overlapping fluorescent signals and facilitate particle tracking. The ac bias was turned on between the nanoelectrodes of the nanoprobe placed near a target nanobead, enabling the trapping of the nanobead near the nanoprobe tip. The nanobead was manipulated by maneuvering the nanoprobes via a micromanipulator while keeping the DEP on. To release the nanobead from the nanoprobe tip, the DEP was turned off, causing the trapped nanobead to diffuse away quickly into the solution. Particle tracking was performed via Fiji software and MATLAB 2020b.

### DEP trapping and manipulation of isolated mitochondria

The mitochondria of the HeLa cells were isolated using the Cell Mitochondria Isolation Kit (Beyotime Institute of Biotechnology, Shanghai, China). Briefly, the cells were pelleted at 300*g* and washed with PBS. The cells were homogenized 25 times with a glass homogenizer in mitochondrial extraction reagent (provided in the kit). The suspensions were subsequently centrifuged at 1000*g* for 10 min, after which the supernatants were collected. The resulting supernatants were further centrifuged at 11,000*g* for 10 min to isolate the mitochondria. The isolated mitochondria were resuspended in storage buffer from the kit, maintained on ice, and used for subsequent experiments.

MitoTracker Green FM at a final concentration of 500 nM was added to the mitochondrial sample and incubated at 37°C with gentle shaking for 15 min. The sample was then centrifuged (11,000*g*) for 10 min, resulting in a mitochondrial pellet. The pellet was washed with PBS and centrifuged again, after which the supernatant was removed. The resulting mitochondrial pellet was resuspended in the following solution (consisting of 250 mM sucrose, 1 mM F108, and 10 mM Hepes, pH adjusted to 7.4 with KOH and sterile-filtered to 0.2 μm) for DEP experiments.

The mitochondria were trapped at the nanoprobe tip by activating the ac bias (frequency of 1 MHz and peak-to-peak voltage of 7.0 V). The release of the target from the nanoprobe tip was achieved by turning off the DEP.

### Intracellular mitochondrial biopsy

The cells were imaged using inverted fluorescence microscopy and an LSM 980 inverted confocal laser scanning microscope (Zeiss, Germany). An ac voltage was applied to the two faces of the probe via a function generator to create an electric field at the nanoprobe tip (frequency of 1 MHz). The mitochondria were trapped by the electric field gradient to the tip of the nanoprobe. After holding the nanoprobe tip inside the cell for 60 s, the tip was slowly retracted from the cell. The presence of a fluorescence spot at the tip after retraction confirmed the successful extraction of the mitochondria.

During the mitochondrial biopsy success rate experiment (Fig. 4I and fig. S22), the success rate is defined as the percentage of successful mitochondrial extractions under specified voltage and insertion duration conditions. Successful extraction was confirmed by observing fluorescent dots at the nanoprobe tip after DEP manipulation (no fluorescence = failed extraction).

### Isolation of template DNA and qPCR analysis

The extraction of DNA was performed using the TaKaRa MiniBEST Universal Genomic DNA Extraction Kit Ver. 5.0 in accordance with the manufacturer’s protocol. All the qPCR experiments were carried out using an ABI 7500 Real-Time PCR System (USA) in optical qPCR tubes. The qPCR primer pairs used for amplification were designed through Primer-BLAST and obtained from Sangon Biotech Co. Ltd. (table S1 lists the primers used in this study). The isolated mitochondria were transferred into a qPCR tube that contained 8.8 μl of diethyl pyrocarbonate water, 10 μl of SYBR Premix Ex Taq (Tli RNase H Plus) (2× concentration), 0.4 μl of ROX Reference Dye II (50× concentration) (TaKaRa), and 0.4 μl each of the forward, and reverse primers were added. All the samples were analyzed using the following protocol: initial preheating at 95°C for 3 min, followed by 55 cycles of 95°C for 15 s and 60°C for 35 s. The fluorescence data were recorded at the end of each annealing/extension step. Melting peak analysis was performed by gradually increasing the temperature from 60° to 95°C at a rate of 0.5°C/s to validate the qPCR results. The sequences of the qPCR products were analyzed using Sanger sequencing.

### Mitochondrial transplantation between living cells

mt-DsRed–HeLa and mt-GFP–HeLa cells were seeded 24 hours before the experiments were performed onto 35-mm tissue culture–treated dishes inside two-well culture inserts (ibidi). For the experiments, the culture medium was replaced with CO_2_-independent growth medium supplemented with 10% FBS (Thermo Fisher Scientific) and 1% penicillin-streptomycin (Thermo Fisher Scientific). The nanoprobe is mounted on an LSM 980 inverted confocal laser scanning microscope equipped with a temperature-controlled incubation chamber.

Before the nanoprobe was inserted into the mt-DsRed–HeLa cells, an ac voltage was applied to both faces of the nanoprobe via a function generator, generating an electric field at the tip of the nanoprobe. The electric field gradient trapped the mitochondria at the tip of the nanoprobe. Following a 60-s pause with the nanoprobe tip inside the cell, the tip was gradually withdrawn. The fluorescence spot at the tip upon retraction confirmed the successful extraction of the mitochondria.

The activation of the electric field in the nanoprobe is maintained to ensure that the trapped mitochondria remain at the tip. The nanoprobe is subsequently inserted into mt-GFP–HeLa cells. After the tip enters the cell, the electric field is deactivated, and the nanoprobe is retracted, leaving the mitochondria within the mt-GFP–HeLa cells, thus completing the process of mitochondrial transfer.

## Supplementary Materials

### The PDF file includes:

Notes S1 to S5 Figs. S1 to S26 Tables S1 to S4 Legend for movie S1 References
