# Supplementary material for: Label-free robotic mitochondrial biopsy
Source: Sci Adv. 2025 Oct 22;11(43):eadx4289. doi: 10.1126/sciadv.adx4289 (PMC12542936; doi:10.1126/sciadv.adx4289)
Supplement: Supplementary file 1 — Notes S1 to S5 Figs. S1 to S26 Tables S1 to S4 Legend for movie S1 References [file sciadv.adx4289_sm.pdf]

Science Advances
AAAS

Supplementary Materials for

Label-free robotic mitochondrial biopsy

Yanmei Ma *et al.*

Corresponding author: Hongri Gu, hongrigu@ust.hk; Chengzhi Hu, hucz@sustech.edu.cn

Sci. Adv. **11**, eadx4289 (2025)
DOI: 10.1126/sciadv.adx4289

The PDF file includes:

Notes S1 to S5
Figs. S1 to S26
Tables S1 to S4
Legend for movie S1
References

Other Supplementary Material for this manuscript includes the following:

Movie S1

Notes 1. Details of the numerical simulation.

1.1 Nanoprobe tip structure.

The interelectrode distance on the nanoprobe tip was set to 2-20 nm.

1.2 Simulation of the dielectrophoresis force.

The dielectrophoresis (DEP) trapping force ($\boldsymbol{F_{DEP}}$) acting on the mitochondria is derived by integrating the Maxwell stress tensor $\boldsymbol{T}$ over the mitochondria surface, formulated as:

$$\boldsymbol{F_{DEP}} = \int_A (\boldsymbol{T} \cdot \boldsymbol{n})\, \mathrm{dA}$$

where $\boldsymbol{n}$ is the unit normal vector to the mitochondrion model surface *(20)*. The surface stress distribution $\boldsymbol{T}$ and resultant $\boldsymbol{F_{DEP}}$ were numerically simulated using finite element software COMSOL Multiphysics with the AC/DC Module. The geometry model consists of a nanoprobe with a 380 nm outer tip diameter and a mitochondrion model (approximated as a homogeneous particle to simplify calculations) with a 1000 nm outer diameter, as shown in Fig. S8. The probe features three layers: a hollow nanopipette innermost layer, an elliptical platinum (Pt) layer divided by a 2 nm gap into two independent electrodes for electric field generation, and an outermost Al_2O_3 insulating layer. Simulations applying a 7 V amplitude, 1 MHz voltage yielded the DEP force versus mitochondrion-tip distance (Fig. 1G), showing rapid force decay with increasing mitochondrion-tip separation. Additionally, Fig. S9 illustrates the magnitude of the electric field gradient $\nabla|\boldsymbol{E}|^2$ (proportional to the DEP force) near the nanoprobe tip and mitochondrion at a 100 nm separation distance between the mitochondrion and the nanoprobe tip.

1.3 Double-shell model of mitochondria.

To further investigate the motion characteristics of mitochondria driven by dielectrophoretic forces, we also analyzed the Clausius-Mossotti (CM) factor based on the double-shell model to observe the changes in the direction of dielectrophoretic forces on mitochondria across different voltage frequencies. $\mathrm{Re}[f_{CM}]$ indicates the real part of the CM factor defined as:

$$\mathrm{Re}[f_{CM}(\omega)] = \left(\frac{\varepsilon_{eff}^* - \varepsilon_m^*}{\varepsilon_{eff}^* + 2\varepsilon_m^*}\right)$$

where ε_m^* is the frequency-dependent complex permittivity of the surrounding medium, which is given as

$$\varepsilon_m^* = \varepsilon_m - \mathrm{j}\frac{\sigma_m}{\omega}$$

where σ_m is the medium conductivity, ω indicates the frequency and $\mathrm{j} = \sqrt{-1}$. In addition, ε_{eff}^* is the effective complex permittivity of the particle with a membrane, which can be expressed as:

$$\varepsilon_{eff}^* = \varepsilon_s^* \frac{\left(\frac{r_2}{r_1}\right)^3 + 2\frac{\varepsilon_p^* - \varepsilon_s^*}{\varepsilon_p^* + 2\varepsilon_s^*}}{\left(\frac{r_2}{r_1}\right)^3 - \frac{\varepsilon_p^* - \varepsilon_s^*}{\varepsilon_p^* + 2\varepsilon_s^*}}$$

where ε_s^* and ε_p^* denote the complex permittivity of the membrane and particle core, respectively. r_1 and r_2 refer to the radii of the particle core and whole particle, respectively. If the "particle core" still contains more than two substances, such as a membrane, the ε_p^* can be further extended as ε_{eff}^* to determine the CM factor of the multishell model. Thus, the CM factor of mitochondria exhibiting a double-shell dielectric model can be calculated to accurately

describe the dielectric properties based on the specific parameters (Fig. S20). The CM factor curve of mitochondria is plotted in Fig. 4D, which illustrates that the trapping force based on positive DEP can be achieved within the frequency range of 1.26-214.66 MHz. A frequency of 1 MHz was selected in the experiment to obtain a sufficiently strong DEP force.

Notes 2. Details of the Péclet number calculation.

The Péclet number (*Pe*) quantifies the relative importance of convective versus diffusive transport (*21*). When $Pe >> 1$, convection dominates, whereas $Pe << 1$ suggests that diffusion is the dominant process. Mitochondrial trapping involves both fluidic diffusion and DEP-induced convection driven by the DEP force, constituting a mass transfer model. *Pe* is defined as:

$$Pe = \frac{Lu}{D}$$

where L is the characteristic length given by the mitochondrial diameter (1 μm), u is the velocity of the mitochondrion driven by the DEP force, and D is the mass diffusion coefficient. The velocity u changes with the DEP force and is calculated using Stokes' drag law:

$$u = \frac{F_{DEP}}{6\pi\mu r}$$

where F_{DEP} is the DEP force, μ is the dynamic viscosity of the surrounding fluid, and r is the mitochondrial radius. The mass diffusion coefficient D is calculated as:

$$D = \frac{k_B T}{6\pi\mu r}$$

where k_B is the Boltzmann constant (1.38×10^{-23} J/K) and T is the temperature of the mitochondrion/fluid (293.15 K for all calculations).

The DEP force variation with distance is shown in Fig. 1G. Thus, the variation of *Pe* with distance can be obtained (Fig. S10). When the distance between the mitochondrion and the tip is less than 1.8 μm, the *Pe* is greater than 1, indicating that convection due to the DEP force dominates and mitochondrial trapping is achievable. When the distance exceeds 1.8 μm, the *Pe* is less than 1, indicating diffusion dominates and mitochondrial trapping cannot be achieved.

Notes 3. Details of the automation techniques.

As shown in Fig. S12, the key automation techniques include nanoprobe tip positioning (Fig. S13), cell detection (Fig. S14), penetration path optimization (Fig. S15), and cell-tip contact detection (Fig. S16).

3.1 Nanoprobe tip positioning

Nanoprobe tip positioning was accomplished using an improved background subtraction algorithm, whose basic working principle is depicted in Fig. S13 (A). First, the system removes the probe from the field of view to capture the background image with the cells, as shown in Fig. S13 (A) (i). After the background image is acquired, the probe is moved back into the field of view, and an image of the nanoprobe with the background is obtained (Fig. S13 (A) (ii)). By subtracting the background image of the cells from the probe image, a nanoprobe tip image with the cell background filtered out is generated. This image is then processed through binarization, morphological noise removal, contour extraction, and boundary point extraction to finally obtain the tip positions, thereby achieving tip positioning (Fig. S13 (A) (iv)).

3.2 Cell-tip contact detection

Cell-tip contact detection was performed to accurately determine the relative height between the nanoprobe tip and the cell surface, as the height of the cells is heterogeneous. The detailed

procedure is shown in Fig. S16(A)(i-ii). First, a trapezoidal mask is generated based on the nanoprobe tip position to eliminate the influence of tip movement on the detection of cell deformation, as illustrated in Fig. S16(A)(iii). Next, cell deformation is detected using a motion history image (MHI) algorithm as the nanoprobe tip approaches the cell along the Z-axis. The resulting images before and after contact detection are shown in Fig. S16(A)(iv) and Fig. S16(A)(vi), respectively. The contact is deemed successful when the pixel mean value (PMV) obtained from the MHI algorithm exceeds a set threshold of 1, thereby precisely determining the relative position between the nanoprobe tip and the cell surface in the Z direction.

Notes 4. Details of the exponential decay model.

During long-term intracellular ROS/RNS detection (Fig. S17), the cell can be simplified as a sealed chamber with a fixed initial amount of ROS/RNS inside. When the Pt electrode undergoes an oxidation reaction with ROS/RNS, the intracellular ROS/RNS are consumed. This consumption process follows a simple dissipation model, and the specific dissipation process is as follows:

$$Q = nFZ$$

$$Q = It$$

$$I = AC$$

$$C = \frac{n}{V}$$

where:

Q: charge (Coulombs, C)
n: amount of substance (mol)
F: Faraday constant (96485 C/mol)
Z: ratio between the charge transferred and the amount of substance (here, Z=1)
I: current (A)
t: time (s)
A: sensor sensitivity (A/mol L^{-1})
C: concentration (mol/L)
V: volume (L)

Thus:

$$\ln n = -\frac{A}{VFZ}t + C$$

where C is a constant.

Let $e^C = K$ (where K represents the initial amount of n inside the cell); then,

$$n = K \cdot e^{-\frac{A}{VFZ}t}$$

and

$$I = \frac{KA}{V} \cdot e^{-\frac{A}{VFZ}t}$$

which can be simplified as

$$I \sim e^{-\frac{1}{\tau}t}$$

where τ is the time constant, which represents the rate of decay. The relationship between the current and time reveals an exponential decrease in the current. This decay is characterized by the time constant τ, which is influenced by the cell's volume (V) and the sensitivity (A). Based on this model, we calculated the volume of the tested cells.

Notes 5. Details on analyzing the nanoprobe tip heating.

To characterize nanoprobe tip heating, the heating power P_D (active power) dissipated by the DEP nanotweezer during mitochondrial biopsy was experimentally measured at V_{pp} = 7 V and f = 1 MHz. As shown in Fig. S21A, the active power P_D and impedance $\boldsymbol{Z_D}$ of the DEP nanotweezer were determined by connecting a known resistor R (165.5 ohm) in series and measuring the input voltage waveform and the voltage waveform across R using an oscilloscope. The formulas are as follows:

$$P_D = \mathrm{Re}[(\boldsymbol{V_{in}}\text{-}\boldsymbol{V_R})\left(\frac{\boldsymbol{V_R}}{R}\right)^*] \qquad \boldsymbol{Z_D} = \frac{(\boldsymbol{V_{in}}\text{-}\boldsymbol{V_R})R}{\boldsymbol{V_R}}$$

where $\boldsymbol{V_{in}}$ and $\boldsymbol{V_R}$ represent the root mean square (RMS) values of the input voltage and the voltage across R, respectively, including both amplitude and phase information. The operations *Re[A]* and *(A)** denote taking the real part of ***A*** and the complex conjugate of ***A***, respectively. As illustrated in Fig. S21B and C, the active power P_D and impedance magnitude $|\boldsymbol{Z_D}|$ vary with the insertion length L of the nanoprobe tip immersed in PBS solution. The impedance magnitude decreases as L increases, while the power shows a progressive rise with greater insertion depths. When L = 2365 μm, the nanoprobe tip reaches the position required for cell penetration, and the active power P_D is less than 0.3 mW.

Coupling the experimentally measured P_D (0.3 mW) with COMSOL thermal simulations allows for the quantification of the temperature increase around the nanoprobe tip. The geometric model used in the COMSOL simulation is shown in Fig. S21D and consists of a 2000-μm-long nanoprobe tip and a 4000-μm-diameter spherical PBS solution (thermal conductivity: 0.6 W/(m·K)). Since there is no externally driven fluid movement in the cell culture dish, the heat flux condition on the surface of the PBS sphere was set as external natural convection. After assigning the power P_D as the boundary heat source on the probe surface, the temperature distribution near the nanoprobe tip (Fig. S21E) and the temperature change curve within 10 μm of the tip (Fig. S21F) were obtained. The results indicate a temperature rise of less than 0.04 K at the nanoprobe tip relative to the ambient temperature (293.15 K), confirming that the DEP nanotweezer induces no adverse thermal effects on cells. It is important to note that 0.04 K represents the maximum theoretical temperature rise, not only because the total power of the DEP nanotweezer was applied to the 2000-μm-long nanoprobe tip, but also due to the fact that heat transfer within the PBS region was limited solely to conduction, leading to a higher temperature increase.

Supplementary figures

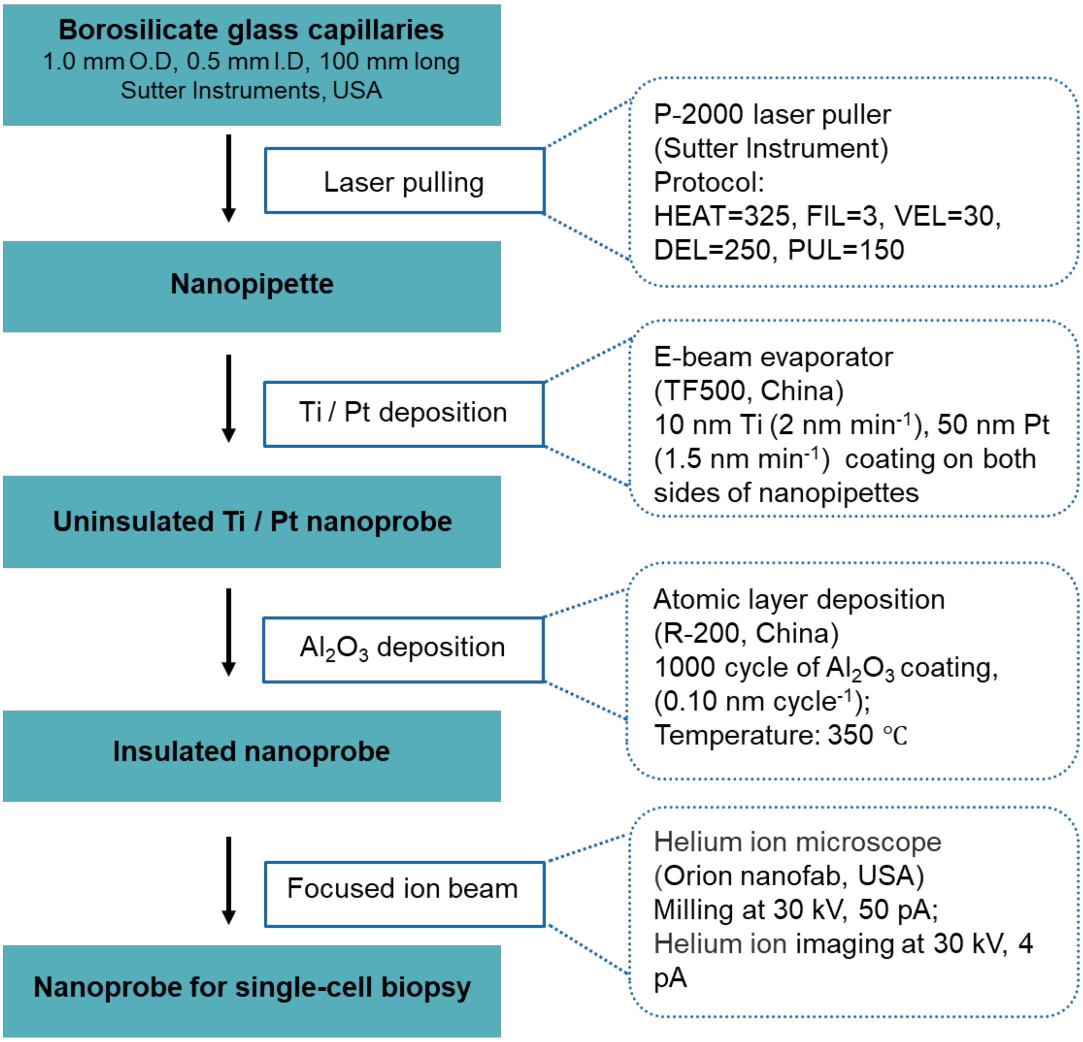

Fig. S1. Nanoprobe fabrication process. Briefly, glass capillaries were first pulled to create sharp nanopipettes. Ti and Pt layers were coated on two sides of the pulled nanopipettes. An insulating layer of Al_2O_3 was then applied to the Ti/Pt nanoprobe. The final tip of the insulated nanoprobe was milled with an Focused Ion Beam (FIB) to expose the Pt layer, resulting in the production of a multifunctional nanoprobe. The final tip size of the nanoprobe is determined by the position of FIB milling.

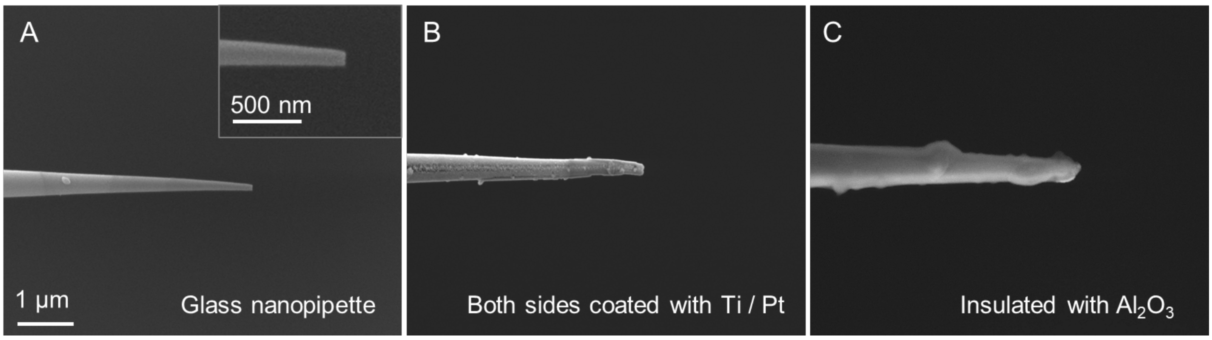

Fig. S2. SEM images of the nanoprobe after different fabrication steps. (A) The glass nanopipette was fabricated by laser-pulling a glass capillary, with a tip size maintained at ~100 nm (inset is a magnified view of the nanopipette tip). (B) Both sides of the nanopipette were sequentially coated with a 10 nm-thick Ti (adhesion layer) and a 50 nm-thick Pt. The tip diameter increased to ~200 nm, and a nanogap formed between the two sides. (C) The nanoprobe was finally insulated with 100 nm-thick Al_2O_3, resulting in a tip outer diameter of ~400 nm.

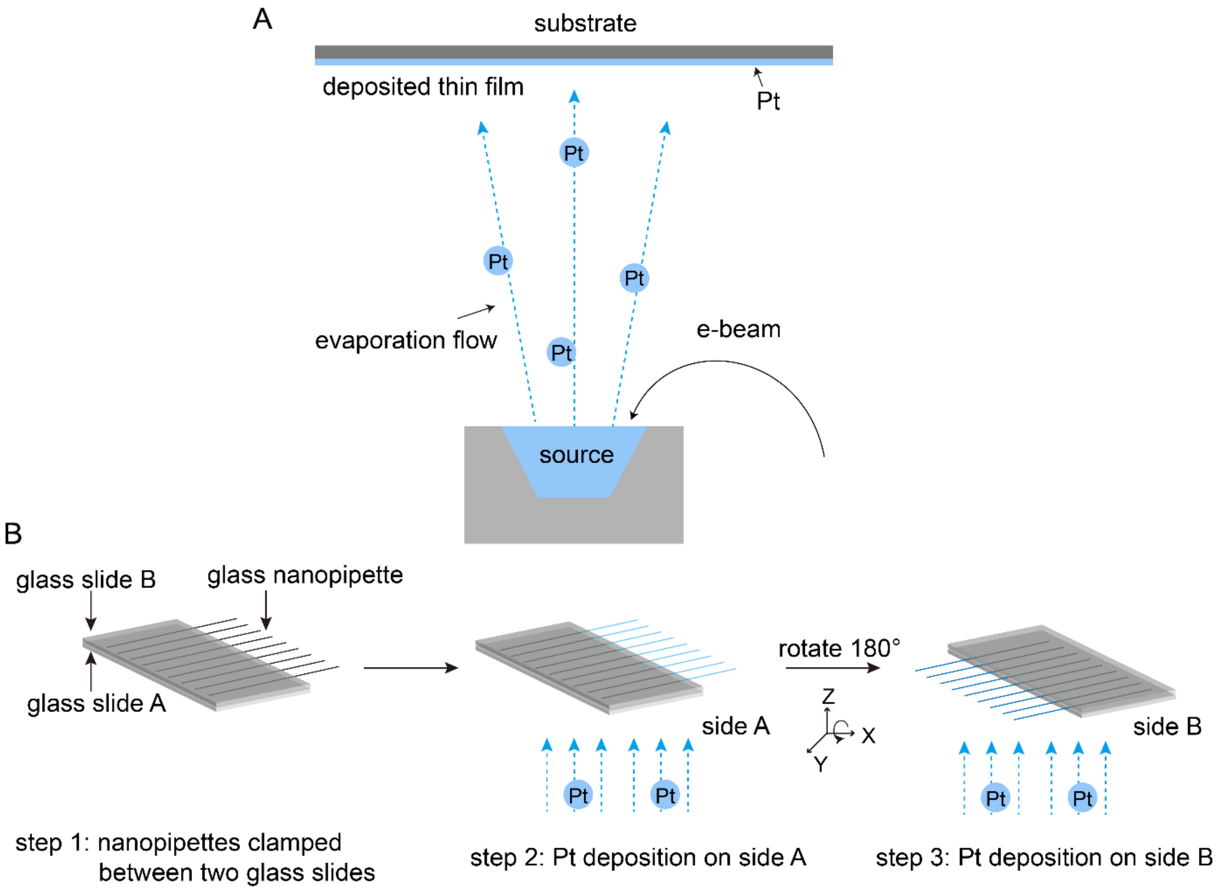

Fig. S3. Schematic diagram of electron-beam evaporation deposition of Pt on both sides of nanopipettes. (A) During the evaporation, a focused electron beam heats a Pt source in a vacuum, rapidly vaporizing it. The Pt atoms then travel freely and deposit onto the substrate, forming a pure and uniform thin film. (B) The glass nanopipette was fixed between two glass slides. After depositing a 50-nm-thick Pt layer on one side, the slide was rotated by 180°, and an additional 50-nm Pt layer was deposited on the opposite side, achieving symmetric Pt coating on both surfaces. Electron-beam evaporation deposition is highly directional. Due to the shadowing effect, this approach reliably forms nanometer-scale insulating gaps in regions

perpendicular to the deposition axis.

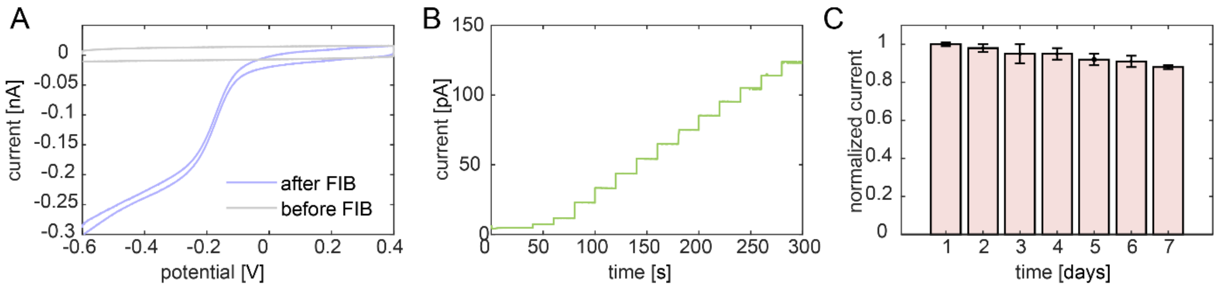

Fig. S4. Electrochemical characterization of the nanoprobe. (A) Cyclic voltammogram of the nanoprobes before and after FIB milling in 2 mM $Ru(NH_3)_6Cl_3$. Scan rate = 100 mV s^{-1}. There was no faradaic current response before FIB milling, indicating the Al_2O_3 insulation layer was effective. After FIB milling, the cathodic current was about 300 pA for 2mM $Ru(NH_3)_6Cl_3$, demonstrating successful exposure of the Pt nanoelectrodes after FIB milling. (B) Current response and corresponding calibration curve with increasing H_2O_2 concentrations (0, 0.05, 0.2, 0.4, 0.6, 0.8, 1.0, 1.2, 1.4, 1.6, 1.8, 2.0, 2.2, 2.4 mM) at an applied potential of +0.85 V (vs. Ag/AgCl). The response current exhibited a concentration-dependent increase, demonstrating a linear correlation between current signal and H_2O_2 concentration. (C) Stability tests of the nanoprobe for 7 days. The nanoprobe was tested under constant H_2O_2 concentration for 7 days, demonstrating excellent stability.

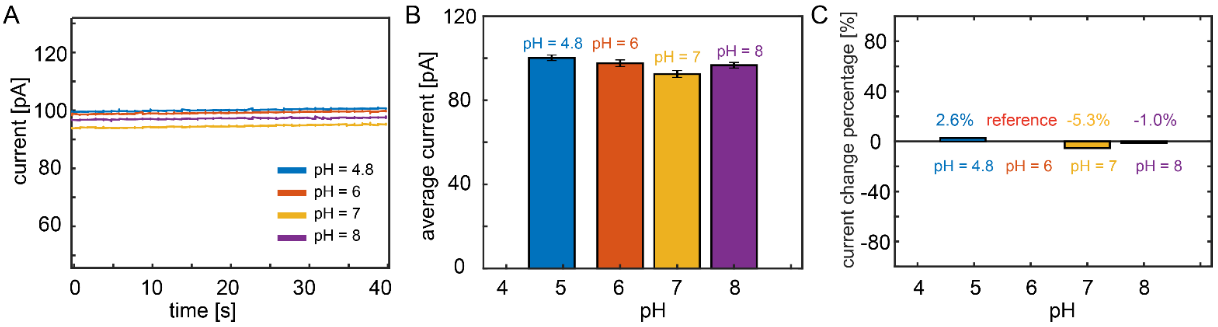

Fig. S5. pH sensitivity test of the nanoprobe. (A) Amperometric responses of the nanoprobe to 2 mM H_2O_2 at pH 4.8, 6.0, 7.0, and 8.0, recorded at +0.85 V (vs. Ag/AgCl). (B) Statistical analysis of the current under different pH conditions. (C) Current variations at pH values of 4.8, 7.0, and 8.0 relative to the reference at pH 6.

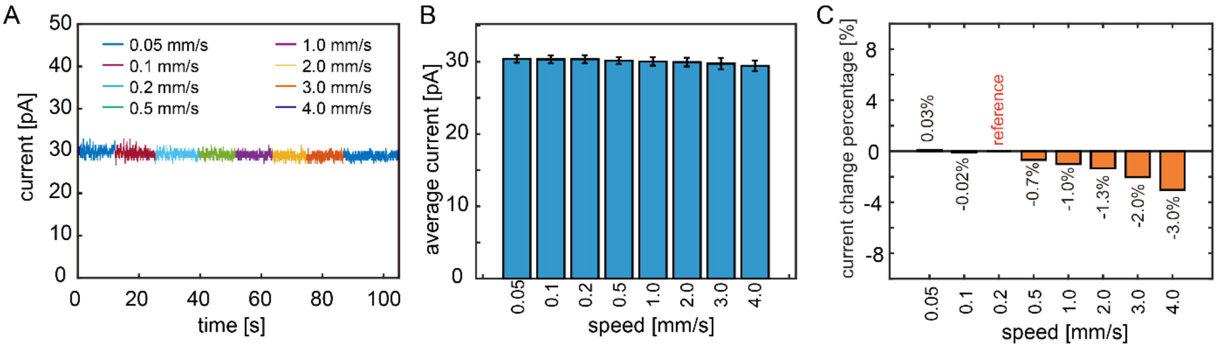

Fig. S6. Stability test of the nanoprobe under different motion speeds. (A) Amperometric responses of the nanoprobe to 1 mM H_2O_2 at speeds of 0.05, 0.1, 0.2, 0.5, 1.0, 2.0, 3.0, and 4.0 mm/s, recorded at +0.85 V (vs. Ag/AgCl). (B) Statistical analysis of the response current at different motion speeds. (C) Current variations at different motion speeds relative to the 0.2 mm/s reference (the speed used in this study).

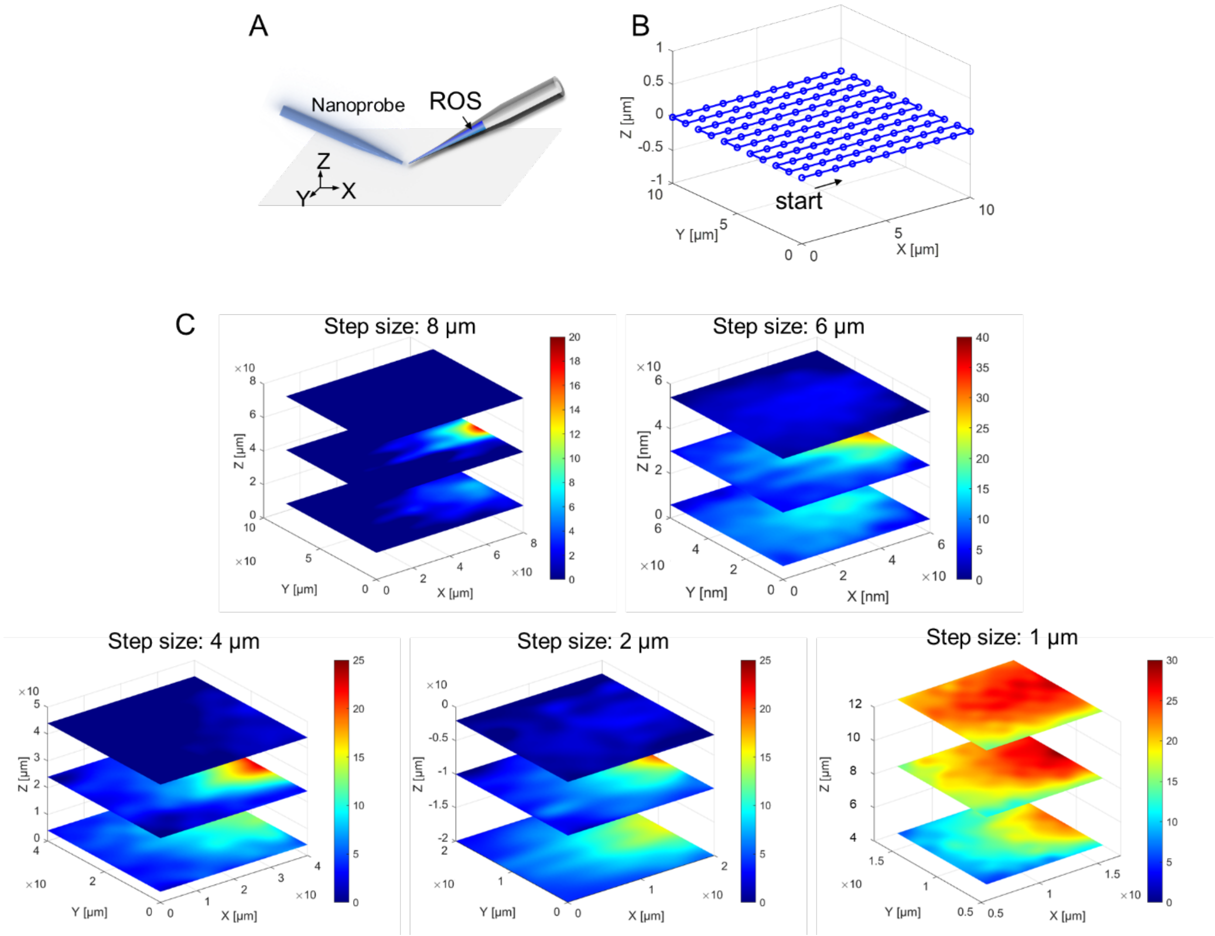

Fig. S7. Spatial resolution measurement of nanoprobe. (A) Schematic of the spatial resolution measurement. A capillary with a ~600 nm diameter tip was fixed on a micromanipulator (µMp-4, Sensapex, Finland) and filled with 1 mM ROS solution, serving as a static ROS source in the absence of driven flow. The nanoprobe, fixed on another micromanipulator (µMp-4, Sensapex, Finland), was moved around the capillary tip to detect surrounding ROS concentrations. Current signals were recorded via an Axopatch Multiclamp 700B low-noise amplifier at a sampling rate of 1 kHz. (B) Trajectory map of nanoprobe movement. (C) Results of the spatial resolution measurement. The nanoprobe was moved in step sizes of 8, 6, 4, 2, and 1 µm, respectively, over 10 steps for each setting, corresponding to total travel distances of 80, 60, 40, 20, and 10 µm, respectively. The color scale represents the current amplitude. Experimental results show the maximum ROS concentration at the center of the ROS-filled capillary tip, with concentration decreasing progressively with increasing distance from the ROS source. By reducing the nanoprobe's step size from 8 µm to 1 µm, the nanoprobe enabled the detection of ROS concentration differences at a 1 µm scale.

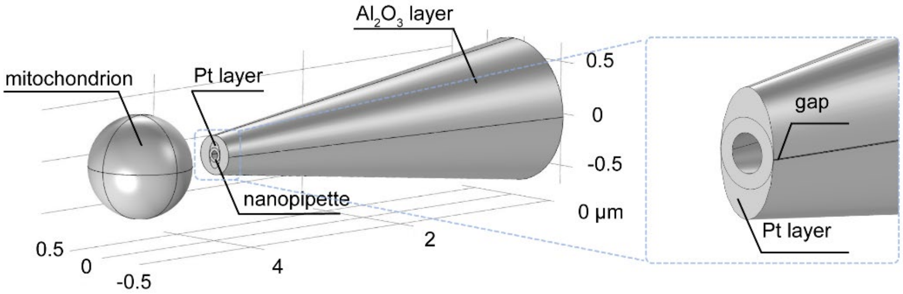

Fig. S8. Geometry of the nanoprobe tip in finite element modeling. The Pt layer of the nanoprobe tip is 50 nm thick, and the Al_2O_3 layer is 100 nm thick. The nanogap between two Pt nanoelectrodes is 2 nm, and the mitochondrion diameter is 1000 nm.

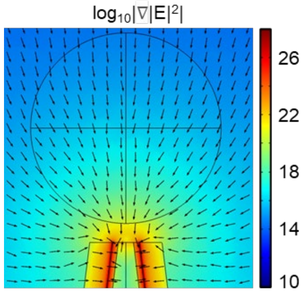

Fig. S9. Simulation result of the electric field gradient ($\nabla|\boldsymbol{E}|^2$). Calculated via a finite element model with a 2 nm nanoelectrode gap, the colormap depicts the distribution of $\nabla|\boldsymbol{E}|^2$, with warm colors (red) indicating higher gradient values. The maximum field gradient ($\nabla|\boldsymbol{E}|^2$) near the gap reaches 10^{28} V^2 m^{-3}.

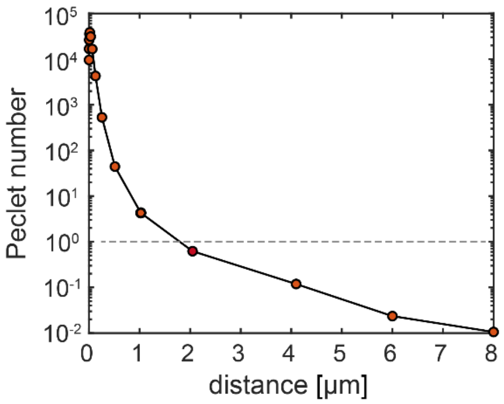

Fig. S10. Péclet number at different mitochondrion-nanoprobe tip distances. The Péclet number (*Pe*) quantifies the relative dominance of convective transport induced by DEP force versus mitochondrial diffusion. A $Pe \gg 1$ indicates that convection dominates, facilitating DEP trapping, whereas $Pe \ll 1$ suggests that diffusion is the dominant process. The result shows that DEP-induced convection achieves effective mitochondrial trapping within an approximate 1.8 μm distance around the tip.

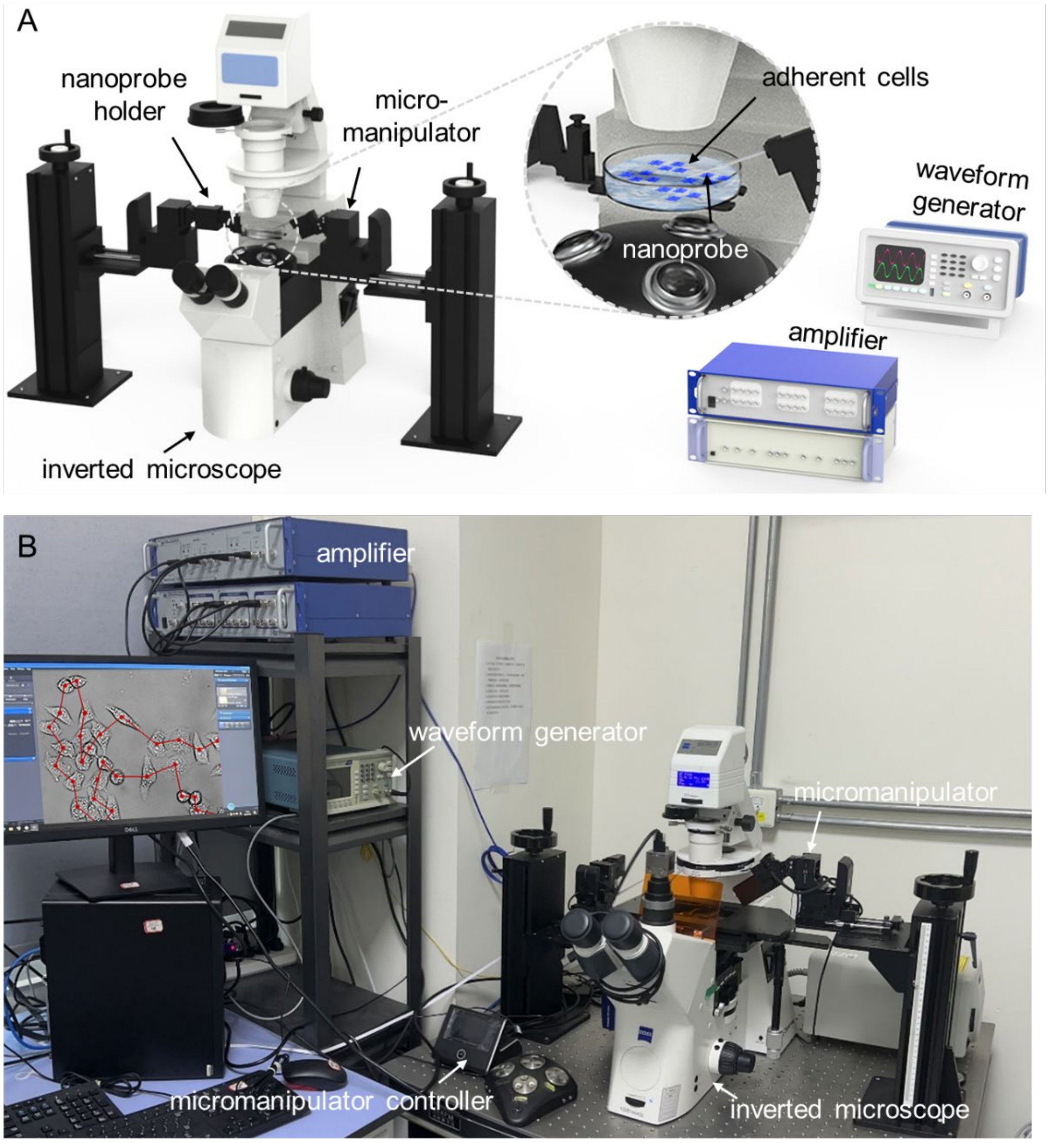

Fig. S11. Robotic micromanipulation system. (A) Schematic of the automatic label-free intracellular mitochondria biopsies using a robotic micromanipulation system. The system employs microscope feedback for cell detection and 3D location of the nanoprobe tip. Micromanipulators control the nanoprobe entry into the cell. An amplifier and waveform generator are used for intracellular electrochemical sensing and dielectrophoretic extraction of mitochondria. (B) A photograph of the robotic micromanipulation system, with the entire setup placed on a vibration isolation platform to minimize mechanical disturbances.

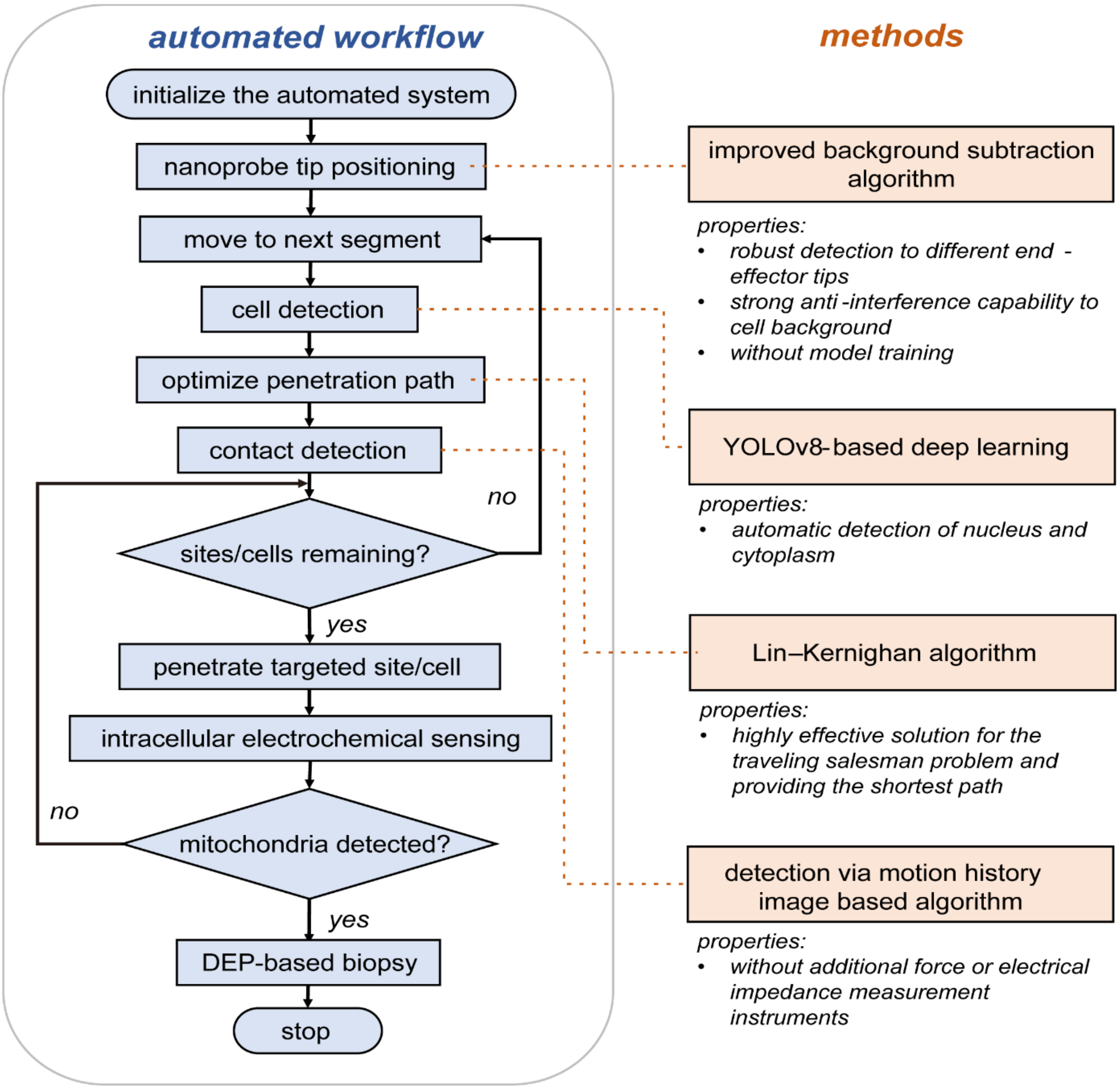

Fig. S12. Workflow of automatic label-free single-cell biopsy.

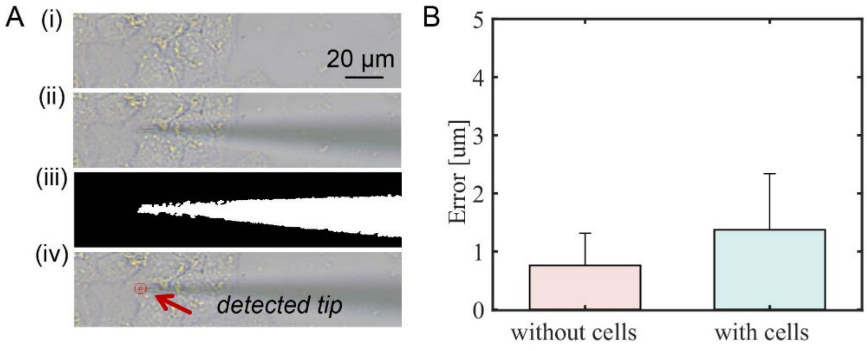

Fig. S13. Nanopipette tip positioning. (A) Detection of the nanoprobe tip. (i) Background image with cells. (ii) Original image of a nanoprobe. (iii) Segmentation of the nanoprobe image. (iv) Nanoprobe tip detection. (B) Nanoprobe tip positioning accuracy under varying background conditions. The mean tip positioning error remains below 2 μm, both in the presence and absence of cellular backgrounds, ensuring precise manipulation of the nanoprobe.

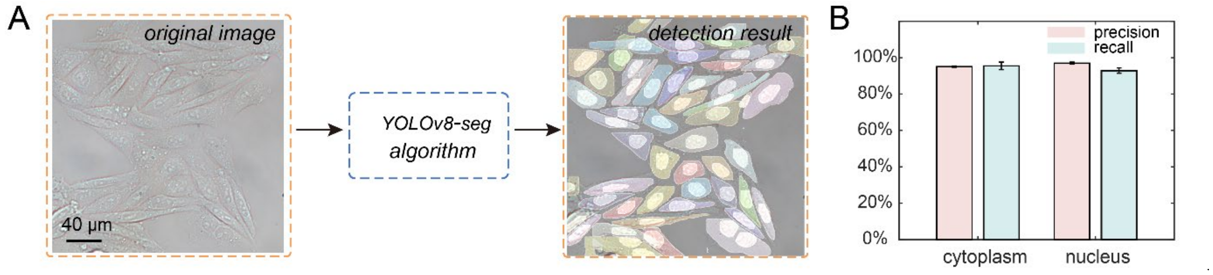

Fig. S14. Cell detection. (A) Cell detection based on the YOLOv8-based deep learning algorithm (22). (B) Quantitative results of cell detection. A dataset of 428 original images, expert-annotated for both cytoplasm and nucleus regions, was utilized for YOLOv8 training. Quantitative analysis demonstrated key segmentation metrics (precision and recall) exceeding 90% for both cytoplasm and nucleus regions. Specifically, precision exceeded 90%, indicating high agreement between positively classified pixels and expert annotations, which minimizes over-segmentation of background regions. Similarly, recall surpassed 90%, demonstrating effective target detection with minimal missed areas. These results collectively establish the algorithm's high segmentation accuracy for cell detection.

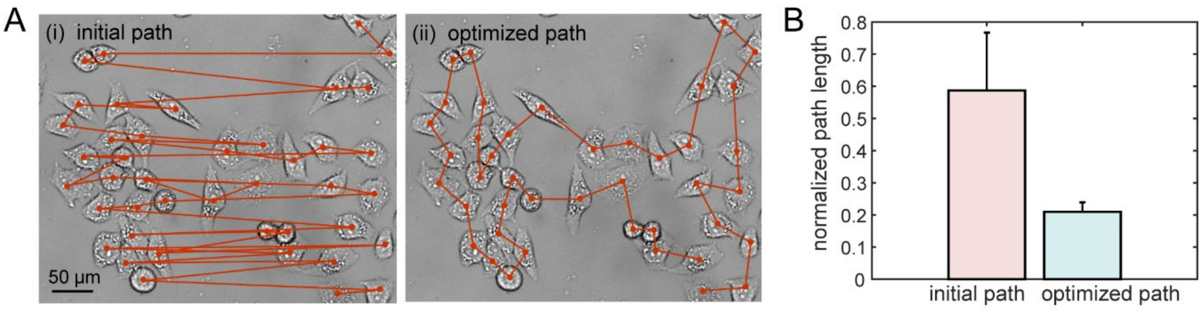

Fig. S15. Penetration path optimization. (A) Penetration path before and after optimization. (i) Initial path. (ii) Optimized path. (B) Quantitative comparison of the penetration path length between the initial path and optimized path. The initial path represents the total length derived from directly sequentially connecting cell detection results (n=10). The optimized path is generated using the Lin-Kernighan algorithm. To quantify the path optimization effect, all path lengths (both initial and post-optimization) were normalized against the maximum value across the ten initial paths. The optimized path is only 35% of the initial path, which enables high-efficiency intracellular biopsies.

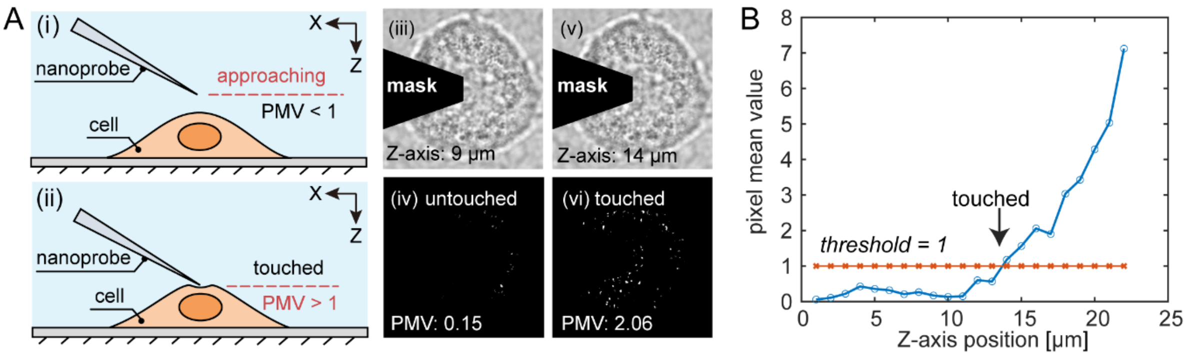

Fig. S16. Cell-tip contact detection. (A) Process of cell-tip contact detection. (i-ii) Schematic diagram showing the nanopipette approaching and touching the cell surface. (iii-iv) Bright-field cell images and images generated by the motion history image (MHI) algorithm before tip-cell contact. (v-vi) Bright-field cell images and MHI images after tip-cell contact. The contact is deemed successful when the pixel mean value (PMV) obtained from the MHI algorithm exceeds a set threshold of 1, thereby precisely determining the relative position between the nanoprobe tip and the cell surface in the Z direction. (B) Quantitative PMV variation versus nanoprobe tip displacement along the Z-axis. Before contact (Z-axis displacement < 10 μm), PMV remains sub-threshold. Upon contact initiation, cellular deformation triggers PMV elevation. Further tip descent amplifies cell deformation, concurrently increasing PMV magnitude, validating threshold-based contact detection feasibility *(23)*.

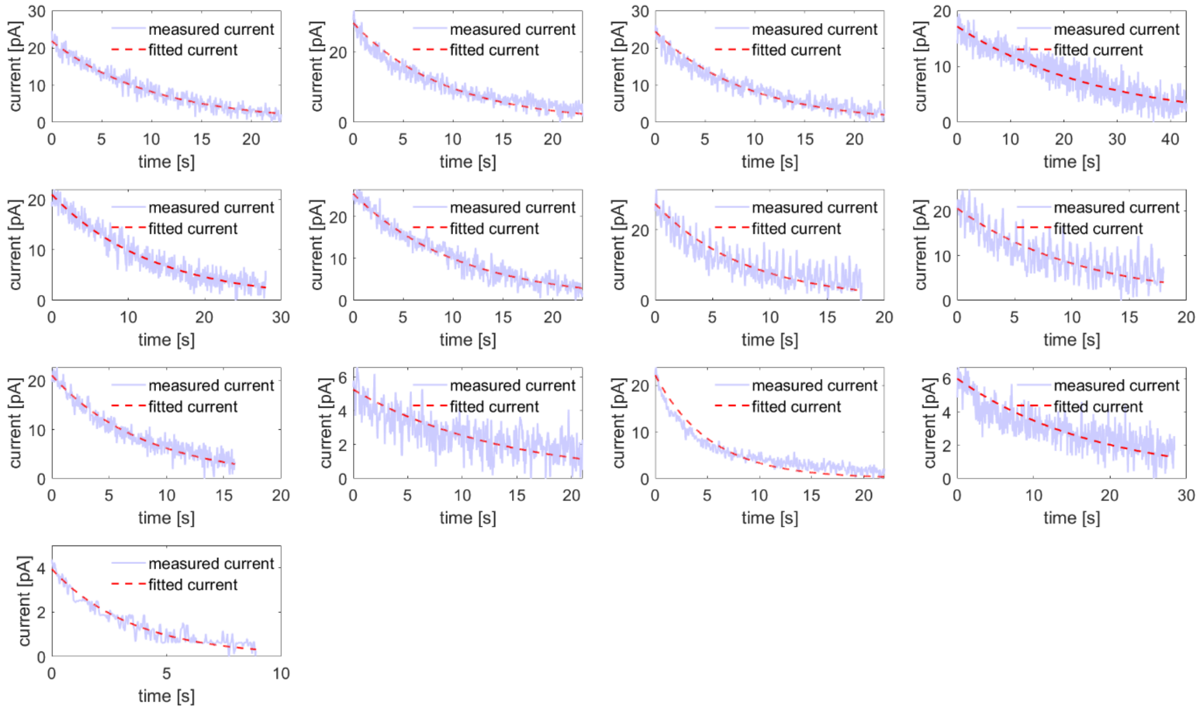

Fig. S17. Amperometric traces obtained from 13 HeLa cells treated with PMA. The light purple line represents the actual measurement data, and the red dashed line represents the fitted curve. These fitted curves were used to construct a decay model (see Note 4), which closely matched our experimental results and enabled the estimation of individual cell volumes.

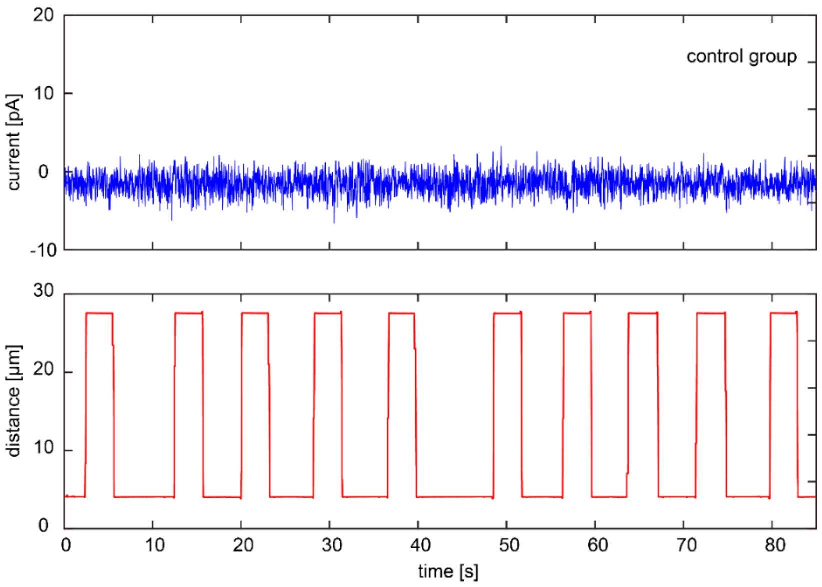

Fig. S18. Detection of intracellular ROS/RNS in untreated cells (control group that does not receive drug treatment). Upper panel: Current measurements during cell penetration using nanoprobe. Lower panel: Corresponding position data of the nanoprobe tip. Untreated cells exhibited negligible signals indistinguishable from background noise. Intracellular ROS levels under physiological conditions were below the detection threshold of our nanoprobe system.

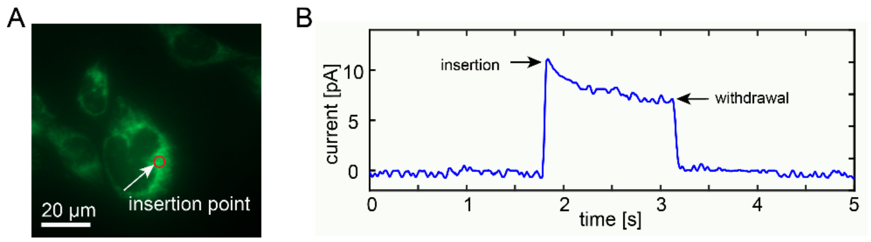

Fig. S19. Mitochondrial fluorescence and corresponding current signal measurement. (A) Mitochondrial fluorescence intensity at the nanoprobe insertion point. (B) Real-time current signals during nanoprobe insertion and withdrawal. The method involved acquiring mitochondrial fluorescence images of cells, followed by the selection of specific penetration sites. Current signals during both penetration and withdrawal were simultaneously recorded, enabling establishment of the correlation between local fluorescence intensity and electrochemical response at each penetration site (see Fig. 3H for results).

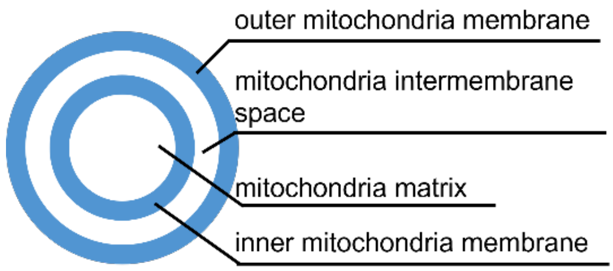

Fig. S20. Theoretical model of the mitochondrion used for CM factor analysis. The double-shell dielectric model of mitochondria represents mitochondria as a double-layered structure consisting of an outer membrane and an inner membrane with an intermembrane space between them. The model assumes that the mitochondria behave like a dielectric material, meaning that they can store and release electric charge. The dielectric properties of the outer and inner membranes are different, which allows for the storage of charge between the two membranes.

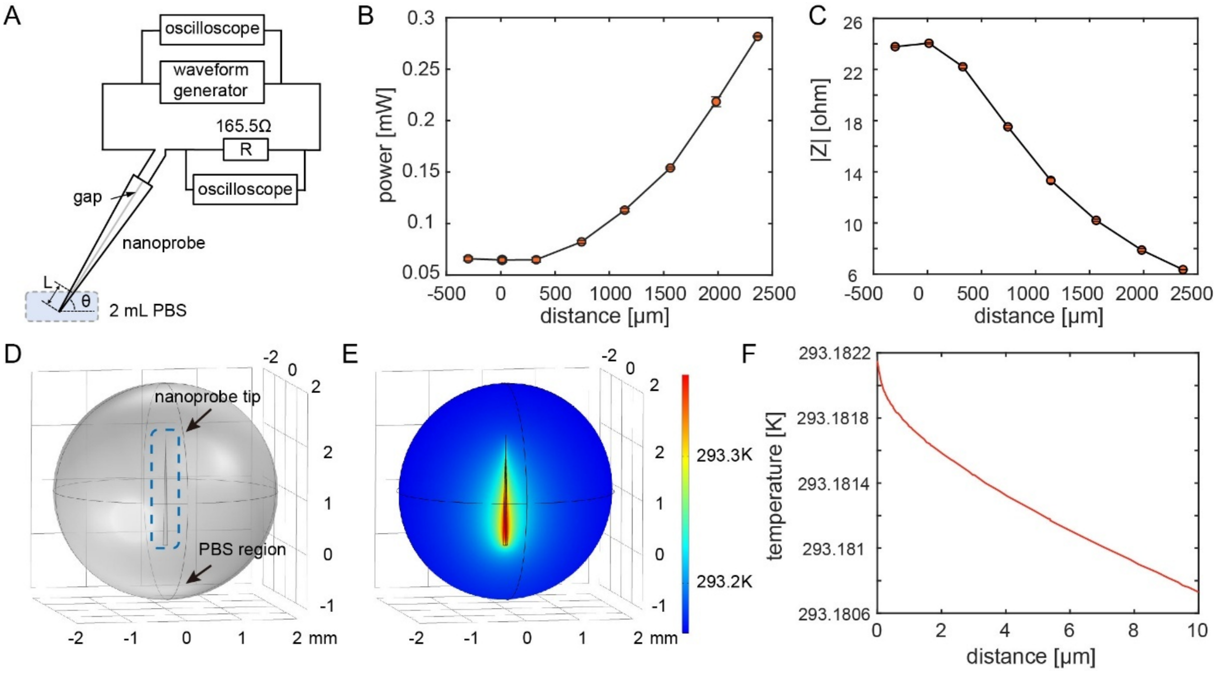

Fig. S21. Power and temperature distribution of DEP nanotweezer. (A) Schematic diagram of the impedance and power measurement for the nanoprobe. (B) The active power delivered to the nanoprobe increases with increasing immersion depth of the nanoprobe tip in PBS. (C) The impedance magnitude of the nanoprobe decreases with increasing immersion depth of the nanoprobe tip in PBS. (D) Geometric model for finite element analysis of the nanoprobe tip. (E) Finite element model of the temperature distribution around the nanoprobe tip. (F) Simulated axial temperature profile at different distances (0-10 μm) from the nanoprobe tip. The results indicate a temperature rise of less than 0.04 K at the nanoprobe tip relative to the ambient temperature (293.15 K), confirming that the DEP nanotweezer induces no adverse thermal effects on cells.

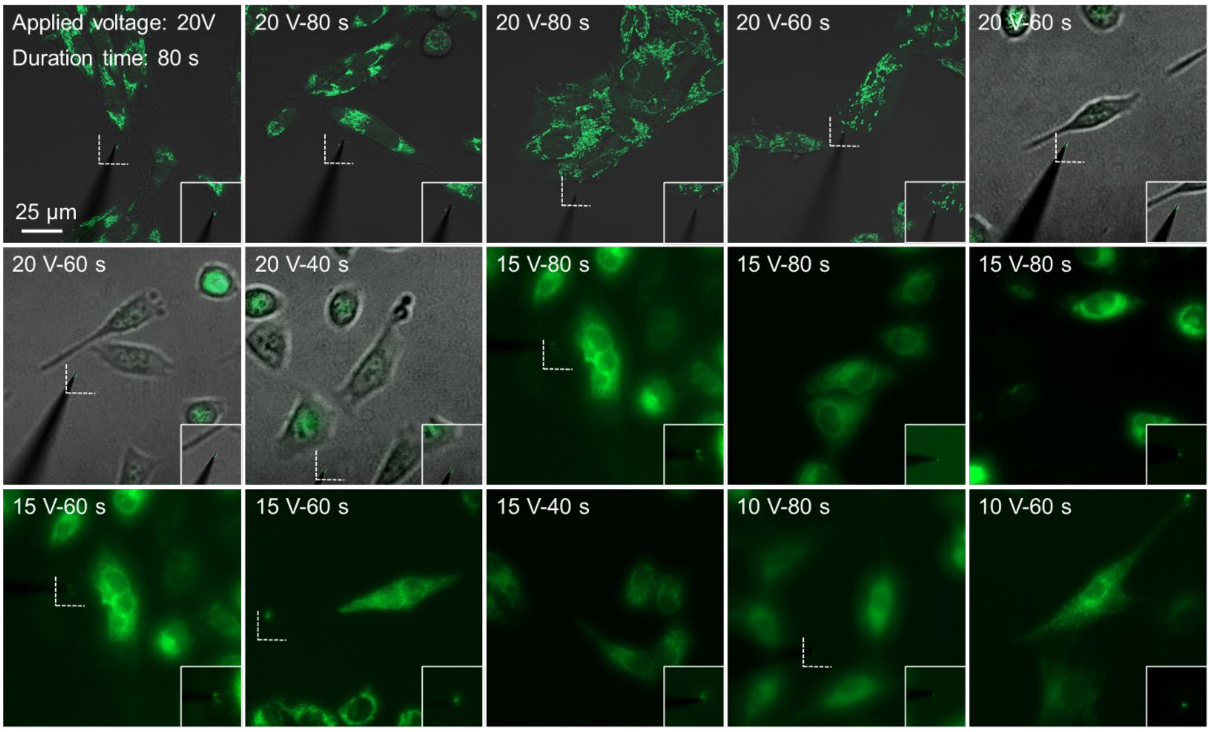

Fig. S22. Fluorescence micrographs of cells following DEP mitochondrial biopsy at different voltages and waiting times. Representative fluorescence images corresponding to the data points in Fig. 4I demonstrate the successful extraction of mitochondria via DEP manipulation. Fluorescent dots (indicating mitochondria labeled with MitoTracker Green) are observed at the nanoprobe tip, confirming the success of the DEP-based isolation. The experiments were performed under varying conditions: applied voltages of 20 V, 15 V, and 10 V, and nanoprobe insertion durations of 80 s, 60 s, and 40 s. A magnified view of the nanoprobe tip (inset) highlights the presence of mitochondria.

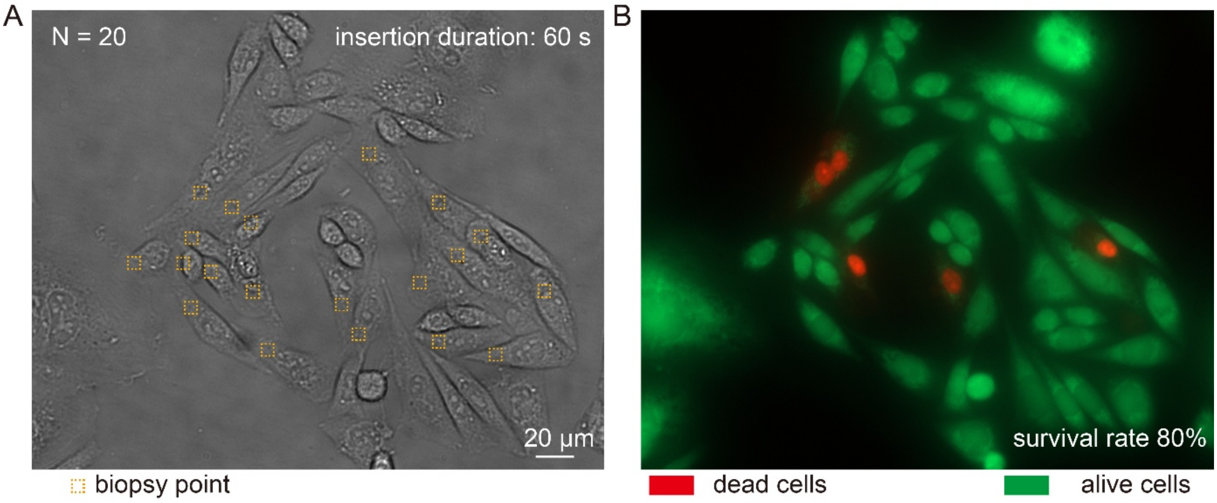

Fig. S23. Cell viability assessment following DEP activation. (A) Bright-field image and (B) fluorescence image of HeLa cells stained with calcein-AM (green, live cells) and propidium iodide (PI; red, dead cells). Cells were first incubated with calcein-AM to label viable cells. A dielectrophoretic (DEP) force (7 V, 1 MHz) was then applied, and the nanoprobe was inserted into individual cells for 60 s (n = 20). After the experiment, PI was added to stain dead cells. The result shows that 80% of cells remained viable following DEP manipulation.

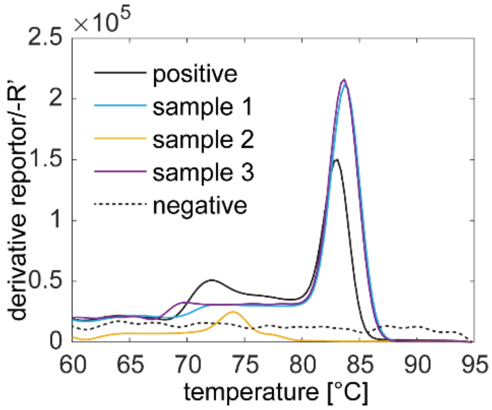

Fig. S24. Melting curves obtained from the qPCR of the DEP-trapped mitochondria. DEP sample groups and the positive control showed a melting peak at the same temperature, confirming successful amplification. The corresponding amplification curves are shown in Fig. 5F.

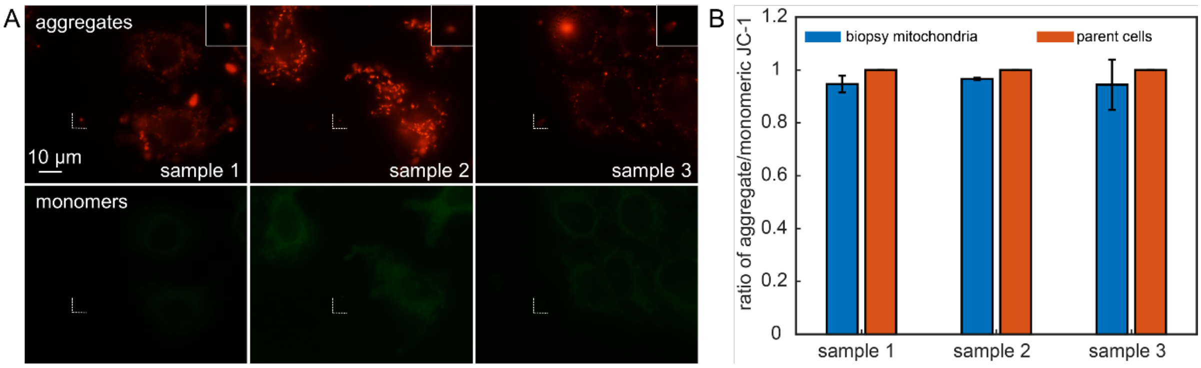

Fig. S25. Assessment of mitochondrial membrane potential in biopsy samples. (A) JC-1 staining was performed before biopsy. The fluorescence intensity of both the biopsied mitochondria and the parent cells was measured. Inset is an enlarged view of the biopsied mitochondria. (B) JC-1 fluorescence ratio (red/green) in biopsy mitochondria and corresponding parent cells. JC-1 red fluorescence represents mitochondria with high membrane potential, whereas green fluorescence reflects monomeric JC-1 in mitochondria whose membrane potential has decreased (depolarized). The sampled mitochondria exhibited clear red fluorescence, with intensity comparable to that of the parent cells from which they were sampled. These results indicate that the biopsied mitochondria maintained J-aggregate formation, confirming their normal polarization state.

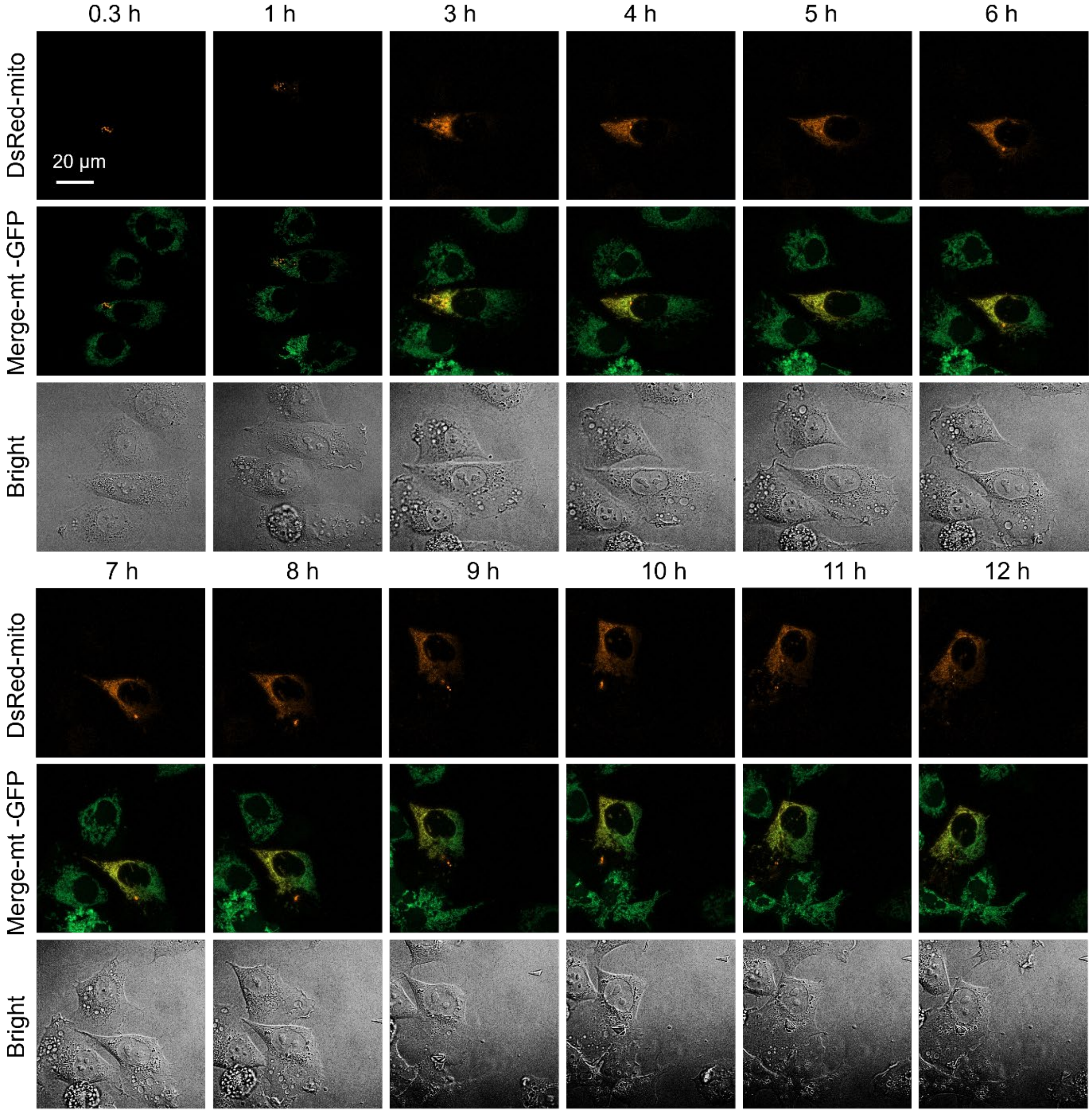

Fig. S26. Microscopy image of recipient cells after mitochondrial transfer at different time points. Fluorescence and bright images of the recipient cell at various time points post-transplantation demonstrated the gradual integration of DsRed-Mito into the mt-GFP-HeLa cell mitochondrial network. Red fluorescent mitochondria were extracted from a donor cell (mt-DsRed-HeLa), and the recipient cell was a mt-GFP-HeLa. In this case, total red fluorescence increased over time. We hypothesize that this phenomenon may originate from co-transferred mRNA during transplantation, enabling temporary mt-DsRed translation in the recipient cell.

Table S1. Performance comparison of the ROS sensors developed in this study with existing intracellular ROS sensors.

Sensitivity (A/mol)	Range	Reference
277.45×10^{-9}	0.1-1 mM	(14)
26×10^{-9}	0.001-1 mM	(49)
278×10^{-9}	0.08-1.28 mM	(50)
3.7×10^{-9}	1nM-1mM	(51)
-	0.5-5 mM	(52)
-	0.005-1 mM	(53)
-	0.08-0.8 mM	(54)
-	0.05-1 mM	(55)
**54.09×10^{-9}**	**0.05-2.4 mM**	**This work**

Table S2. Performance comparison of the DEP nanotweezers developed in this study with other reported intracellular DEP nanotweezers.

Size	$\nabla \lvert E \rvert^2$	Target	Reference
100 nm	10^{28} V^2 m^{-3}	DNA/RNA/Mito	(8)
300 nm	10^{24} V^2 m^{-3}	RNA	(56)
200 nm	10^8 V/m	DNA	(57)
100 nm	10^{28} V^2 m^{-3}	RNA	(58)
**500 nm**	**10^{28} V^2 m^{-3}**	**Mito**	**This work**

Table S3. Performance comparison of different automatic microrobotic systems for adherent cells.

Success rate %	Survival rate %	Reference
53.3	95.8	(59)
60.3	82	(60)
43.75	95.74	(61)
95.2	97.2	(23)
82.4	95.7	(62)
58.5	81.5	(63)
60.9	57.7	(64)
**68.1**	**96**	**This work**

Table S4. Primers used in qPCR for the amplification of mitochondrial DNA.

Gene	Forward primer	Reverse primer
MT-ND1	5'-ACGCCATAAAACTCTTCACCAAAG-3'	5'-GGGTTCATAGTAGAAGAGCGATGG-3'

Movie S1 (.mp4 format). Large-scale single-cell probing with an automated robotic system.

REFERENCES AND NOTES

1. R. Hooke, *Micrographia: Or Some Physiological Descriptions of Minute Bodies Made by Magnifying Glasses, with Observations and Inquiries Thereupon* (Dover Publications, 2003).

2. B. S. Erwin Neher, The patch clamp technique. *Sci. Am.* **266**, 44–51 (1992).

3. A. Huxley, From overshoot to voltage clamp. *Trends Neurosci.* **25**, 553–558 (2002).

4. T. DiTommaso, J. M. Cole, L. Cassereau, J. A. Buggé, J. L. S. Hanson, D. T. Bridgen, B. D. Stokes, S. M. Loughhead, B. A. Beutel, J. B. Gilbert, K. Nussbaum, A. Sorrentino, J. Toggweiler, T. Schmidt, G. Gyuelveszi, H. Bernstein, A. Sharei, Cell engineering with microfluidic squeezing preserves functionality of primary immune cells in vivo. *Proc. Natl. Acad. Sci. U.S.A.* **115**, E10907–E10914 (2018).

5. W. Dou, G. Shan, Q. Zhao, M. Malhi, A. Jiang, Z. Zhang, A. González-Guerra, S. Fu, J. Law, R. M. Hamilton, J. A. Bernal, X. Liu, Y. Sun, J. T. Maynes, Robotic manipulation of cardiomyocytes to identify gap junction modifiers for arrhythmogenic cardiomyopathy. *Sci. Robot.* **9**, eadm8233 (2024).

6. J. Liu, J. Wen, Z. R. Zhang, H. J. Liu, Y. Sun, Voyage inside the cell: Microsystems and nanoengineering for intracellular measurement and manipulation. *Microsyst. Nanoeng.* **1**, 15020 (2015).

7. A. Shakoor, W. Gao, L. Zhao, Z. Jiang, D. Sun, Advanced tools and methods for single-cell surgery. *Microsyst. Nanoeng.* **8**, 47 (2022).

8. B. P. Nadappuram, P. Cadinu, A. Barik, A. J. Ainscough, M. J. Devine, M. Kang, J. Gonzalez-Garcia, J. T. Kittler, K. R. Willison, R. Vilar, P. Actis, B. Wojciak-Stothard, S.-H. Oh, A. P. Ivanov, J. B. Edel, Nanoscale tweezers for single-cell biopsies. *Nat. Nanotechnol.* **14**, 80–88 (2018).

9. M. Aramesh, C. Forró, L. Dorwling-Carter, I. Lüchtefeld, T. Schlotter, S. J. Ihle, I. Shorubalko, V. Hosseini, D. Momotenko, T. Zambelli, E. Klotzsch, J. Vörös, Localized detection of ions

and biomolecules with a force-controlled scanning nanopore microscope. *Nat. Nanotechnol.* **14**, 791–798 (2019).

10. W. Chen, O. Guillaume-Gentil, P. Y. Rainer, C. G. Gäbelein, W. Saelens, V. Gardeux, A. Klaeger, R. Dainese, M. Zachara, T. Zambelli, J. A. Vorholt, B. Deplancke, Live-seq enables temporal transcriptomic recording of single cells. *Nature* **608**, 733–740 (2022).

11. J. Li, W. D. Jamieson, P. Dimitriou, W. Xu, P. Rohde, B. Martinac, M. Baker, B. W. Drinkwater, O. K. Castell, D. A. Barrow, Building programmable multicompartment artificial cells incorporating remotely activated protein channels using microfluidics and acoustic levitation. *Nat. Commun.* **13**, 4125 (2022).

12. B. Mandracchia, X. Hua, C. Guo, J. Son, T. Urner, S. Jia, Fast and accurate sCMOS noise correction for fluorescence microscopy. *Nat. Commun.* **11**, 94 (2020).

13. H. Wang, G. Han, H. Tang, R. Zhang, Z. Liu, Y. Sun, B. Liu, J. Geng, Z. Zhang, Synchronous photoactivation-imaging fluorophores break limitations of photobleaching and phototoxicity in live-cell microscopy. *Anal. Chem.* **95**, 16243–16250 (2023).

14. X.-W. Zhang, Q.-F. Qiu, H. Jiang, F.-L. Zhang, Y.-L. Liu, C. Amatore, W.-H. Huang, Real-time intracellular measurements of ROS and RNS in living cells with single core–shell nanowire electrodes. *Angew. Chem. Int. Ed. Engl.* **56**, 12997–13000 (2017).

15. R. Li, M. Li, J. Qiu, K. Li, Y. Liu, C. Cui, Precise robotic picking up of polar body for biopsy application. *IEEE Trans Autom Sci Eng* **22**, 5895–5905 (2024).

16. R. Elnathan, M. G. Barbato, X. Guo, A. Mariano, Z. Wang, F. Santoro, P. Shi, N. H. Voelcker, X. Xie, J. L. Young, Y. Zhao, W. Zhao, C. Chiappini, Biointerface design for vertical nanoprobes. *Nat. Rev. Mater.* **7**, 953–973 (2022).

17. W. Kim, J. K. Ng, M. E. Kunitake, B. R. Conklin, P. Yang, Interfacing silicon nanowires with mammalian cells. *J. Am. Chem. Soc.* **129**, 7228–7229 (2007).

18. A. Shakoor, M. Xie, T. Luo, J. Hou, Y. Shen, J. K. Mills, Achieving automated organelle biopsy on small single cells using a cell surgery robotic system. *IEEE Trans. Biomed. Eng.* **66**, 2210–2222 (2019).

19. D. B. Zorov, M. Juhaszova, S. J. Sollott, Mitochondrial reactive oxygen species (ROS) and ROS-induced ROS release. *Physiol. Rev.* **94**, 909–950 (2014).

20. X. Wang, X.-B. Wang, P. Gascoyne, General expressions for dielectrophoretic force and electrorotational torque derived using the Maxwell stress tensor method. *J. Electrostat.* **39**, 277–295 (1997).

21. B. E. Rapp, "Chapter 9 - Fluids," in *Microfluidics: Modeling, Mechanics and Mathematics* (Elsevier, 2017), pp. 243–263.

22. G. Jocher, A. Chaurasia, J. Qiu, Ultralytics YOLO, Version 8.0.0, GitHub (2023); https://ultralytics.com.

23. J. Liu, V. Siragam, Z. Gong, J. Chen, M. D. Fridman, C. Leung, Z. Lu, C. Ru, S. Xie, J. Luo, R. M. Hamilton, Y. Sun, Robotic adherent cell injection for characterizing cell-cell communication. *IEEE Trans. Biomed. Eng.* **62**, 119–125 (2015).

24. J. Song, C.-H. Xu, S. Huang, W. Lei, Y. Ruan, H. Lu, W. Zhao, J.-J. Xu, H.-Y. Chen, Ultrasmall nanopipette: Toward continuous monitoring of redox metabolism at subcellular level. *Angew. Chem. Int. Ed. Engl.* **57**, 13226–13230 (2018).

25. D. Kim, J. Luo, E. A. Arriaga, A. Ros, Deterministic ratchet for sub-micrometer (bio)particle separation. *Anal. Chem.* **90**, 4370–4379 (2018).

26. A. Barik, X. Chen, S. Oh, Ultralow-power electronic trapping of nanoparticles with sub-10 nm gold nanogap electrodes. *Nano Lett.* **16**, 6317–6324 (2016).

27. K. J. Freedman, L. M. Otto, A. P. Ivanov, A. Barik, S.-H. Oh, J. B. Edel, Nanopore sensing at ultra-low concentrations using single-molecule dielectrophoretic trapping. *Nat. Commun.* **7**, 10217 (2016).

28. S. Djafarzadeh, S. M. Jakob, Isolation of intact mitochondria from skeletal muscle by differential centrifugation for high-resolution respirometry measurements. *J. Vis. Exp.* **121**, e55251 (2017).

29. R. Lin, G. Im, A. Luo, Y. Zhu, X. Hong, J. Neumeyer, H. Tang, N. Perrimon, J. M. Melero-Martin, Mitochondrial transfer mediates endothelial cell engraftment through mitophagy. *Nature* **629**, 660–668 (2024).

30. J. G. Lees, A. M. Kong, Y. C. Chen, P. Sivakumaran, D. Hernández, A. Pébay, A. J. Harvey, D. K. Gardner, S. Y. Lim, Mitochondrial fusion by M1 promotes embryoid body cardiac differentiation of human pluripotent stem cells. *Stem Cells Int.* **2019**, 6380135 (2019).

31. H. Ding, K. Liu, X. Zhao, B. Su, D. Jiang, Thermoelectric nanofluidics probing thermal heterogeneity inside single cells. *J. Am. Chem. Soc.* **145**, 22433–22441 (2023).

32. M. Y. Wen, Y. T. Qi, Y. T. Jiao, X. W. Zhang, W. H. Huang, Reference-attached pH nanosensor for accurately monitoring the rapid kinetics of intracellular H^+ oscillations. *Small* **21**, e2406796 (2025).

33. D. Son, S. Y. Park, B. Kim, J. T. Koh, T. H. Kim, S. An, D. Jang, G. T. Kim, W. Jhe, S. Hong, Nanoneedle transistor-based sensors for the selective detection of intracellular calcium ions. *ACS Nano* **5**, 3888–3895 (2011).

34. H. Zhou, W. Yao, X. Zhou, S. Dong, R. Wang, Z. Guo, W. Li, C. Qin, L. Xiao, S. Jia, Z. Wu, S. Li, Accurate visualization of metabolic aberrations in cancer cells by temperature mapping with quantum coherence modulation microscopy. *ACS Nano* **17**, 8433–8441 (2023).

35. J. R. Casey, S. Grinstein, J. Orlowski, Sensors and regulators of intracellular pH. *Nat. Rev. Mol. Cell Biol.* **11**, 50–61 (2010).

36. R. Bagur, G. Hajnóczky, Intracellular Ca^{2+} sensing: Its role in calcium homeostasis and signaling. *Mol. Cell* **66**, 780–788 (2017).

37. A. Barbot, H. Tan, M. Power, F. Seichepine, G.-Z. Yang, Floating magnetic microrobots for fiber functionalization. *Sci. Robot.* **4**, eaax8336 (2019).

38. Y. Li, F. Xu, J. Qiao, P. Yuan, Into the microscale: Low-input sequencing technologies and applications in medicine. *Innov. Med.* **1**, 100041 (2023).

39. R. Qi, E. Sammler, C. P. Gonzalez-Hunt, I. Barraza, N. Pena, J. P. Rouanet, Y. Naaldijk, S. Goodson, M. Fuzzati, F. Blandini, K. I. Erickson, A. M. Weinstein, M. W. Lutz, J. B. Kwok, G. M. Halliday, N. Dzamko, S. Padmanabhan, R. N. Alcalay, C. Waters, P. Hogarth, T. Simuni, D. Smith, C. Marras, F. Tonelli, D. R. Alessi, A. B. West, S. Shiva, S. Hilfiker, L. H. Sanders, A blood-based marker of mitochondrial DNA damage in Parkinson's disease. *Sci. Transl. Med.* **15**, eabo1557 (2023).

40. T. I. Lima, P.-P. Laurila, M. Wohlwend, J. D. Morel, L. J. E. Goeminne, H. Li, M. Romani, X. Li, C.-M. Oh, D. Park, S. Rodríguez-López, J. Ivanisevic, H. Gallart-Ayala, B. Crisol, F. Delort, S. Batonnet-Pichon, L. R. Silveira, L. S. P. V. Venkata, A. K. Padala, S. Jain, J. Auwerx, Inhibiting de novo ceramide synthesis restores mitochondrial and protein homeostasis in muscle aging. *Sci. Transl. Med.* **15**, eade6509 (2023).

41. Y. S. Hong, S. L. Battle, W. Shi, D. Puiu, V. Pillalamarri, J. Xie, N. Pankratz, N. J. Lake, M. Lek, J. I. Rotter, S. S. Rich, C. Kooperberg, A. P. Reiner, P. L. Auer, N. Heard-Costa, C. Liu, M. Lai, J. M. Murabito, D. Levy, M. L. Grove, A. Alonso, R. Gibbs, S. Dugan-Perez, L. P. Gondek, E. Guallar, D. E. Arking, Deleterious heteroplasmic mitochondrial mutations are associated with an increased risk of overall and cancer-specific mortality. *Nat. Commun.* **14**, 6113 (2023).

42. S. Bannwarth, V. Procaccio, A. S. Lebre, C. Jardel, A. Chaussenot, C. Hoarau, H. Maoulida, N. Charrier, X. Gai, H. M. Xie, M. Ferre, K. Fragaki, G. Hardy, B. M. de Camaret, S. Marlin, C. M. Dhaenens, A. Slama, C. Rocher, J. P. Bonnefont, A. Rötig, N. Aoutil, M. Gilleron, V. Desquiret-Dumas, P. Reynier, J. Ceresuela, L. Jonard, A. Devos, C. Espil-Taris, D. Martinez, P. Gaignard, K.-H. L. Q. Sang, P. Amati-Bonneau, M. J. Falk, C. Florentz, B. Chabrol, I. Durand-Zaleski, V. Paquis-Flucklinger, Prevalence of rare mitochondrial DNA mutations in mitochondrial disorders. *J. Med. Genet.* **50**, 704–714 (2013).

43. L. Yang, Q. Long, J. Liu, H. Tang, Y. Li, F. Bao, D. Qin, D. Pei, X. Liu, Mitochondrial fusion provides an 'initial metabolic complementation'controlled by mtDNA. *Cell. Mol. Life Sci.* **72**, 2585–2598 (2015).

44. M. P. King, G. J. S. Attardi, Human cells lacking mtDNA: Repopulation with exogenous mitochondria by complementation. *Science* **246**, 500–503 (1989).

45. D. Narendra, A. Tanaka, D.-F. Suen, R. J. Youle, Parkin is recruited selectively to impaired mitochondria and promotes their autophagy. *J. Cell Biol.* **183**, 795–803 (2008).

46. F. Bao, L. Zhou, R. Zhou, Q. Huang, J. Chen, S. Zeng, Y. Wu, L. Yang, S. Qian, M. Wang, X. He, S. Liang, J. Qi, G. Xiang, Q. Long, J. Guo, Z. Ying, Y. Zhou, Q. Zhao, J. Zhang, D. Zhang, W. Sun, M. Gao, H. Wu, Y. Zhao, J. Nie, M. Li, Q. Chen, J. Chen, X. Zhang, G. Pan, H. Zhang, M. Li, M. Tian, X. Liu, Mitolysosome exocytosis, a mitophagy-independent mitochondrial quality control in flunarizine-induced parkinsonism-like symptoms. *Sci. Adv.* **8**, eabk2376 (2022).

47. N. Borcherding, J. R. Brestoff, The power and potential of mitochondria transfer. *Nature* **623**, 283–291 (2023).

48. T. Huang, T. Zhang, X. Jiang, A. Li, Y. Su, Q. Bian, H. Wu, R. Lin, N. Li, H. Cao, D. Ling, J. Wang, Y. Tabata, Z. Gu, J. Gao, Iron oxide nanoparticles augment the intercellular mitochondrial transfer–mediated therapy. *Sci. Adv.* **7**, eabj0534 (2021).

49. Y. Jiao, Y. R. Kang, M. Y. Wen, H. Q. Wu, X. W. Zhang, W. H. Huang, Fast antioxidation kinetics of glutathione intracellularly monitored by a dual-wire nanosensor. *Angew. Chem. Int. Ed. Engl.* **62**, e202313612 (2023).

50. Y. Ma, W. Hu, J. Hu, M. Ruan, J. Hu, M. Yang, Y. Zhang, H. Xie, C. Hu, Bifunctional nanoprobe for simultaneous detection of intracellular reactive oxygen species and temperature in single cells. *Microsyst. Nanoeng.* **10**, 171 (2024).

51. K. Liu, R. Liu, D. Wang, R. Pan, H.-Y. Chen, D. Jiang, Spatial analysis of reactive oxygen species in a 3D cell model using a sensitive nanocavity electrode. *Anal. Chem.* **94**, 13287–13292 (2022).

52. Y.-F. Ruan, F.-Z. Chen, Y.-T. Xu, T.-Y. Zhang, S.-Y. Yu, W.-W. Zhao, D. Jiang, H.-Y. Chen, J.-J. Xu, An integrated photoelectrochemical nanotool for intracellular drug delivery and evaluation of treatment effect. *Angew. Chem. Int. Ed. Engl.* **60**, 25762–25765 (2021).

53. Y.-T. Jiao, H. Jiang, W.-T. Wu, Y.-T. Qi, M.-Y. Wen, X.-K. Yang, Y.-R. Kang, X.-W. Zhang, C. Amatore, W.-H. Huang, Dual-channel nanoelectrochemical sensor for monitoring intracellular ROS and NADH kinetic variations of their concentrations. *Biosens. Bioelectron.* **222**, 114928 (2023).

54. W. Hu, Y. Ma, Z. Zhan, D. Hussain, C. Hu, Robotic intracellular electrochemical sensing for adherent cells. *Cyborg. Bionic. Syst.* **2022**, 9763420 (2022).

55. N. Wang, D. Wang, R. Pan, D. Wang, D. Jiang, H. Y. Chen, H.-Y. Chen, Self-referenced nanopipette for electrochemical analysis of hydrogen peroxide in the nucleus of a single living cell. *Anal. Chem.* **93**, 10744–10749 (2021).

56. X. Li, Y. Tao, D.-H. Lee, H. K. Wickramasinghe, A. P. Lee, In situ mRNA isolation from a microfluidic single-cell array using an external AFM nanoprobe. *Lab Chip* **17**, 1635–1644 (2017).

57. Y. Tao, H. K. Wickramasinghe, Coaxial atomic force microscope probes for dielectrophoresis of DNA under different buffer conditions. *Appl. Phys. Lett.* **110**, 073701 (2017).

58. A. Sahota, B. P. Nadappuram, Z. Kwan, F. Lesept, J. H. Howden, S. Claxton, J. T. Kittler, M. J. Devine, J. B. Edel, A. P. Ivanov, Spatial and temporal single-cell profiling of RNA compartmentalization in neurons with nanotweezers. *ACS Nano* **19**, 18522–18533 (2025).

59. F. Pan, S. Chen, L. Zheng, S. Zhi, X. Chen, D. Sun, A practical micropipette-image calibration method for somatic cell microinjection. *IEEE Trans Autom Sci Eng* **22**, 11955–11967 (2025).

60. F. Pan, Y. Jiao, S. Chen, L. Xing, D. Sun, Deep learning-enhanced dual-module large-throughput microinjection system for adherent cells. *IEEE Trans Autom Sci Eng* **4**, 2409–2422 (2022).

61. F. Pan, S. Chen, Y. Jiao, Z. Guan, A. Shakoor, D. Sun, Automated high-productivity microinjection system for adherent cells. *IEEE Robot. Autom. Lett.* **5**, 1167–1174 (2020).

62. W. Wang, Y. Sun, M. Zhang, R. Anderson, L. Langille, W. Chan, A system for high-speed microinjection of adherent cells. *Rev. Sci. Instrum.* **79**, 104302 (2008).

63. Y. T. Chow, S. Chen, C. Liu, C. Liu, L. Li, C. W. M. Kong, S. H. Cheng, R. A. Li, D. Sun, A high-throughput automated microinjection system for human cells with small size. *IEEE ASME Trans. Mechatron.* **21**, 838–850 (2016).

64. J. Hajduk, K. Szajna, B. Lisowski, Z. Rajfur, The influence of microinjection parameters on cell survival and procedure efficiency. *MethodsX* **10**, 102107 (2023).
